# Surveillance for Violent Deaths — National Violent Death Reporting System, 42 States, the District of Columbia, and Puerto Rico, 2019

**DOI:** 10.15585/mmwr.ss7106a1

**Published:** 2022-05-20

**Authors:** Rebecca F. Wilson, Grace Liu, Bridget H. Lyons, Emiko Petrosky, Dominque D. Harrison, Carter J. Betz, Janet M. Blair

**Affiliations:** ^1^Division of Violence Prevention, National Center for Injury Prevention and Control, CDC; ^2^Oak Ridge Institute for Science and Education, Oak Ridge Associated Universities

## Abstract

**Problem/Condition:**

In 2019, approximately 67,000 persons died of violence-related injuries in the United States. This report summarizes data from CDC’s National Violent Death Reporting System (NVDRS) on violent deaths that occurred in 42 states, the District of Columbia, and Puerto Rico in 2019. Results are reported by sex, age group, race and ethnicity, method of injury, type of location where the injury occurred, circumstances of injury, and other selected characteristics.

**Period Covered:**

2019.

**Description of System:**

NVDRS collects data regarding violent deaths obtained from death certificates, coroner and medical examiner records, and law enforcement reports. This report includes data collected for violent deaths that occurred in 2019. Data were collected from 39 states with statewide data (Alabama, Alaska, Arizona, Colorado, Connecticut, Delaware, Georgia, Hawaii, Indiana, Iowa, Kansas, Kentucky, Louisiana, Maine, Maryland, Massachusetts, Michigan, Minnesota, Missouri, Montana, Nebraska, Nevada, New Hampshire, New Jersey, New Mexico, North Carolina, North Dakota, Ohio, Oklahoma, Oregon, Rhode Island, South Carolina, Utah, Vermont, Virginia, Washington, West Virginia, Wisconsin, and Wyoming), three states with data from counties representing a subset of their population (30 California counties, representing 57% of its population, and 47 Illinois counties and 40 Pennsylvania counties, representing at least 80% of their populations), the District of Columbia, and Puerto Rico. NVDRS collates information for each violent death and links deaths that are related (e.g., multiple homicides, homicide followed by suicide, or multiple suicides) into a single incident.

**Results:**

For 2019, NVDRS collected information on 50,374 fatal incidents involving 51,627 deaths that occurred in 42 states (39 states collecting statewide data, 30 California counties, 47 Illinois counties, and 40 Pennsylvania counties), and the District of Columbia. In addition, information was collected for 831 fatal incidents involving 897 deaths in Puerto Rico. Data for Puerto Rico were analyzed separately. Of the 51,627 deaths, the majority (64.1%) were suicides, followed by homicides (25.1%), deaths of undetermined intent (8.7%), legal intervention deaths (1.4%) (i.e., deaths caused by law enforcement and other persons with legal authority to use deadly force acting in the line of duty, excluding legal executions), and unintentional firearm deaths (<1.0%). The term “legal intervention” is a classification incorporated into the *International Classification of Diseases, Tenth Revision,* and does not denote the lawfulness or legality of the circumstances surrounding a death caused by law enforcement. Demographic patterns and circumstances varied by manner of death. The suicide rate was higher for males than for females. Across all age groups, the suicide rate was highest among adults aged 45–54 years. In addition, non-Hispanic American Indian or Alaska Native (AI/AN) and non-Hispanic White (White) persons had the highest suicide rates among all racial and ethnic groups. Among males, the most common method of injury for suicide was a firearm, whereas poisoning was the most common method of injury among females. Among all suicide victims, suicide was most often preceded by a mental health, intimate partner, or physical health problem or by a recent or impending crisis during the previous or upcoming 2 weeks. The homicide rate was higher for males than for females. Among all homicide victims, the homicide rate was highest among persons aged 20–24 years compared with other age groups. Non-Hispanic Black (Black) males experienced the highest homicide rate of any racial or ethnic group. Among all homicide victims, the most common method of injury was a firearm. When the relationship between a homicide victim and a suspect was known, the suspect was most frequently an acquaintance or friend for male victims and a current or former intimate partner for female victims. Homicide most often was precipitated by an argument or conflict, occurred in conjunction with another crime, or, for female victims, was related to intimate partner violence. Nearly all victims of legal intervention deaths were male, and the legal intervention death rate was highest among men aged 25–29 years. The legal intervention death rate was highest among AI/AN males, followed by Black males. A firearm was used in the majority of legal intervention deaths. When a specific type of crime was known to have precipitated a legal intervention death, the type of crime was most frequently assault or homicide. The three most frequent circumstances reported for legal intervention deaths were as follows: the victim’s death was precipitated by another crime, the victim used a weapon in the incident, and the victim had a mental health or substance use problem (other than alcohol use). Unintentional firearm deaths were most frequently experienced by males, White persons, and persons aged 15–24 years. These deaths most frequently occurred while the shooter was playing with a firearm and were precipitated by a person unintentionally pulling the trigger or mistakenly thinking the firearm was unloaded. The rate of deaths of undetermined intent was highest among males, particularly among Black and AI/AN males, and among adults aged 30–44 years. Poisoning was the most common method of injury in deaths of undetermined intent, and opioids were detected in nearly 80% of decedents tested for those substances.

**Interpretation:**

This report provides a detailed summary of data from NVDRS on violent deaths that occurred in 2019. The suicide rate was highest among AI/AN and White males, whereas the homicide rate was highest among Black males. Mental health problems, intimate partner problems, interpersonal conflicts, and acute life stressors were primary circumstances for multiple types of violent death.

**Public Health Action:**

Violence is preventable, and data can guide public health action. NVDRS data are used to monitor the occurrence of violence-related fatal injuries and assist public health authorities in developing, implementing, and evaluating programs, policies, and practices to reduce and prevent violent deaths. For example, the New Hampshire Violent Death Reporting System (VDRS), Indiana VDRS, and Colorado VDRS have used their VDRS data to guide suicide prevention efforts and generate reports highlighting where additional focus is needed. In New Hampshire, VDRS data have been used to monitor the increase in suicide rates during 2014–2018 and guide statewide collaborative prevention efforts. Indiana VDRS used local data to demonstrate differences in suicide and other related mental health problems among Black persons and highlight a need for improved suicide awareness and culturally competent mental health care. The Colorado VDRS conducted geospatial and demographic analysis, considering local VDRS data with existing suicide prevention efforts and resources, to identify regions with high suicide rates regions and populations at high risk for suicide. Similarly, states participating in NVDRS have used their VDRS data to examine related to homicide in their state. In North Carolina for example, where homicide rates among AI/AN and Black persons were approximately 2.5 times higher than the statewide homicide rate, the North Carolina VDRS program aims to partner with historically Black colleges and universities in the state to train researchers to use VDRS data to address health equity issues in and around their immediate community.

## Introduction

According to National Vital Statistics System mortality data obtained from CDC’s Web-based Injury Statistics Query and Reporting System (WISQARS),* violence-related injuries led to 67,304 deaths in the United States in 2019 (*1*). Suicide was the 10th leading cause of death overall in the United States and disproportionately affected young and middle-aged populations. By age group, suicide was the second leading cause of death for persons aged 10–34 years and the fourth leading cause of death for adults aged 35–44 years. Non-Hispanic American Indian or Alaska Native (AI/AN) and non-Hispanic White (White) males had the highest rates of suicide compared with all other racial and ethnic groups and females.

In 2019, homicide was the 16th leading cause of death overall in the United States but disproportionately affected young persons (*1*). Homicide was among the five leading causes of death for children aged 1–14 years, the third leading cause of death for persons aged 15–34 years, and the fifth leading cause of death for persons aged 35–44 years. Homicide was the leading cause of death for non-Hispanic Black (Black) males aged 15–34 years and the second leading cause of death for Black boys aged 1–14 years.

Public health authorities require accurate, timely, and complete surveillance data to better understand and ultimately prevent the occurrence of violent deaths in the United States (*2*,*3*). In 2000, in response to an Institute of Medicine^†^ report noting the need for a national fatal intentional injury surveillance system (*4*), CDC began planning to implement the National Violent Death Reporting System (NVDRS) (*2*). The goals of NVDRS are to

- collect and analyze timely, high-quality data for monitoring the magnitude and characteristics of violent deaths at national, state, and local levels;
- ensure data are disseminated routinely and expeditiously to public health officials, law enforcement officials, policymakers, and the public;
- ensure data are used to develop, implement, and evaluate programs and strategies that are intended to reduce and prevent violent deaths and injuries at national, state, and local levels; and
- build and strengthen partnerships among organizations and communities at national, state, and local levels to ensure that data are collected and used to reduce and prevent violent deaths and injuries.

NVDRS is a state-based active surveillance system that collects data on the characteristics and circumstances associated with violence-related deaths in participating states, the District of Columbia, and Puerto Rico (*2*). Deaths collected by NVDRS include suicides, homicides, legal intervention deaths (i.e., deaths caused by law enforcement acting in the line of duty and other persons with legal authority to use deadly force, excluding legal executions), unintentional firearm deaths, and deaths of undetermined intent that might have been due to violence.^§^ The term “legal intervention” is a classification incorporated into the *International Classification of Diseases, Tenth Revision* (*ICD-10)* (*5*) and does not denote the lawfulness or legality of the circumstances surrounding a death caused by law enforcement.

Before implementation of NVDRS, single data sources (i.e., death certificates) provided only limited information and few circumstances from which to understand patterns of violent deaths. NVDRS filled this surveillance gap by providing more detailed information. NVDRS is the first system to 1) provide detailed information on circumstances precipitating violent deaths, 2) link multiple source documents so that each incident can contribute to the study of patterns of violent deaths, and 3) link multiple deaths that are related to one another (e.g., multiple homicides, suicide pacts, or homicide followed by suicide of the suspect).

NVDRS data collection began in 2003 with six participating states (Maryland, Massachusetts, New Jersey, Oregon, South Carolina, and Virginia) (Figure). Seven states (Alaska, Colorado, Georgia, North Carolina, Oklahoma, Rhode Island, and Wisconsin) began data collection in 2004, three (Kentucky, New Mexico, and Utah) in 2005, two (Ohio and Michigan) in 2010, and 14 (Arizona, Connecticut, Hawaii, Illinois, Indiana, Iowa, Kansas, Maine, Minnesota, New Hampshire, New York, Pennsylvania, Vermont, and Washington) in 2015. In 2017, eight additional states began data collection (Alabama, California,^¶^ Delaware, Louisiana, Missouri, Nebraska, Nevada, and West Virginia), along with the District of Columbia and Puerto Rico. NVDRS received funding in 2018 for a nationwide expansion that included the remaining 10 states (Arkansas, Florida, Idaho, Mississippi, Montana, North Dakota, South Dakota, Tennessee, Texas, and Wyoming), which began data collection in 2019. Since 2018, CDC has provided NVDRS funding to all 50 states, the District of Columbia, and Puerto Rico. NVDRS data are updated annually and are available to the public through CDC’s WISQARS at https://www.cdc.gov/injury/wisqars/nvdrs.html. Case-level NVDRS data are available to interested researchers who meet eligibility requirements via the NVDRS Restricted Access Database (https://www.cdc.gov/violenceprevention/datasources/nvdrs/dataaccess.html).

**FIGURE:**
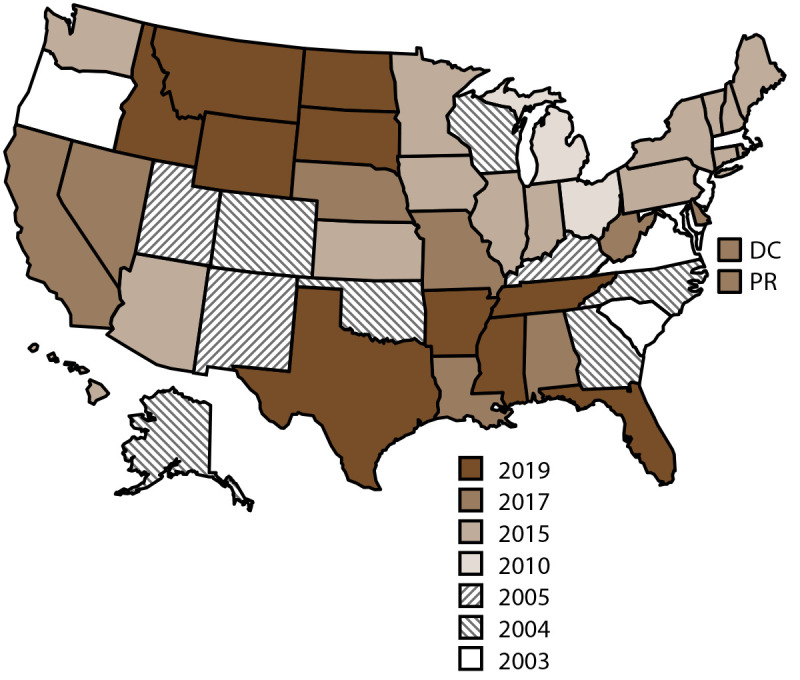
States participating in the National Violent Death Reporting System, by year of initial data collection* — United States and Puerto Rico, 2003–2019 **Abbreviations:** DC = District of Columbia; NVDRS = National Violent Death Reporting System; PR = Puerto Rico. * Map of United States indicates the year in which the state or territory began collecting data in NVDRS. California began collecting data for a subset of violent deaths in 2005 but ended data collection in 2009. In 2017, California collected data from death certificates for all NVDRS cases in the state; data for violent deaths that occurred in four counties (Los Angeles, Sacramento, Shasta, and Siskiyou) also include information from coroner or medical examiner reports and law enforcement reports. In 2018, California collected data from death certificates for all violent deaths in the state in 2018 (n = 6,641); data for violent deaths that occurred in 21 counties (Amador, Butte, Fresno, Humboldt, Imperial, Kern, Kings, Lake, Los Angeles, Marin, Mono, Placer, Sacramento, San Benito, San Mateo, San Diego, San Francisco, Shasta, Siskiyou, Ventura, and Yolo) also included information from coroner or medical examiner reports and law enforcement (n = 3,658; 55.1%). In 2019, California collected data from death certificates for all violent deaths in the state in 2019 (n = 6,586); data for violent deaths that occurred in 30 counties (Amador, Butte, Colusa, Fresno, Glenn, Humboldt, Imperial, Kern, Kings, Lassen, Lake, Los Angeles, Marin, Modoc, Mono, Orange, Placer, Sacramento, San Benito, San Francisco, San Mateo, Santa Cruz, Shasta, Siskiyou, Solano, Sonoma, Tehama, Trinity, Ventura, and Yolo) also included information from coroner or medical examiner reports and law enforcement (n = 3,645; 55.3%). Michigan collected data for a subset of violent deaths during 2010–2013 and collected statewide data beginning in 2014. In 2016, Illinois, Pennsylvania, and Washington began collecting data on violent deaths in a subset of counties that represented at least 80% of all violent deaths in their state or in counties where at least 1,800 violent deaths occurred. Illinois’s 2018 data are for violent deaths that occurred in 28 counties (Adams, Boone, Champaign, Cook, DuPage, Effingham, Fulton, Kane, Kankakee, Kendall, Lake, Lasalle, Livingston, Logan, Macoupin, McDonough, McHenry, McLean, Madison, Peoria, Perry, Rock Island, St. Clair, Sangamon, Tazewell, Vermillion, Will, and Winnebago). Pennsylvania’s 2018 data are for deaths that occurred in 39 counties (Adams, Allegheny, Armstrong, Beaver, Berks, Blair, Bradford, Bucks, Cambria, Carbon, Centre, Chester, Clarion, Clearfield, Clinton, Columbia, Crawford, Dauphin, Delaware, Fayette, Forest, Greene, Indiana, Jefferson, Lackawanna, Lancaster, Lehigh, Luzerne, Monroe, Montgomery, Montour, Northampton, Philadelphia, Schuylkill, Union, Wayne, Westmoreland, Wyoming, and York). Illinois’s 2019 data are for violent deaths that occurred in 47 counties (Adams, Alexander, Bond, Boone, Brown, Bureau, Champaign, Clay, Cook, DeKalb, Douglas, DuPage, Effingham, Fayette, Fulton, Grundy, Henry, Iroquois, Jackson, Jefferson, Kane, Kankakee, Kendall, Lake, Lasalle, Livingston, Logan, Macoupin, McDonough, McHenry, McLean, Madison, Menard, Peoria, Perry, Piatt, Putnam, Rock Island, St. Clair, Sangamon, Schuyler, Stark, Tazewell, Vermilion, Wayne, Will, and Winn). Pennsylvania’s 2019 data are for violent deaths that occurred in 40 counties (Adams, Allegheny, Armstrong, Berks, Blair, Bradford, Bucks, Cameron, Cambria, Carbon, Centre, Chester, Clarion, Clearfield, Clinton, Crawford, Dauphin, Delaware, Erie, Fayette, Forest, Greene, Indiana, Jefferson, Lackawanna, Lancaster, Lehigh, Luzerne, Monroe, Montgomery, Northampton, Philadelphia, Schuylkill, Somerset, Sullivan, Susquehanna, Union, Westmoreland, Wyoming, and York). In 2018, Washington began collecting statewide data for all deaths that occurred in the state in 2018. Beginning in 2019, all 50 U.S. states, DC, and Puerto Rico were participating in the system.

This report summarizes NVDRS data on violent deaths that occurred in 42 states, the District of Columbia, and Puerto Rico in 2019. Thirty-nine states collected statewide data (Alabama, Alaska, Arizona, Colorado, Connecticut, Delaware, Georgia, Hawaii, Indiana, Iowa, Kansas, Kentucky, Louisiana, Maine, Maryland, Massachusetts, Michigan, Minnesota, Missouri, Montana, Nebraska, Nevada, New Hampshire, New Jersey, New Mexico, North Carolina, North Dakota, Ohio, Oklahoma, Oregon, Rhode Island, South Carolina, Utah, Vermont, Virginia, Washington, West Virginia, Wisconsin, and Wyoming), and the three remaining states collected data from a subset of counties in their states (30 California counties, 47 Illinois counties, and 40 Pennsylvania counties). This report of 2019 data includes data for three additional states that met inclusion criteria (Montana, North Dakota, and Wyoming) that were not included in 2018. Data for Arkansas, Florida, Idaho, Mississippi, South Dakota, Tennessee, and Texas were ineligible to be included in this report because they were in an optional pilot year for data collection year 2019 and their approved and funded data collection plan represented <50% of all violent deaths in their state, or their data were ineligible to be included in this report because the data did not meet the completeness threshold for circumstances (see Inclusion Criteria).

## Methods

NVDRS compiles information from three required data sources: death certificates, coroner and medical examiner records, and law enforcement reports (*2*). Some participating Violent Death Reporting System (VDRS) programs might also collect information from secondary data sources (e.g., child fatality review team data, Federal Bureau of Investigation Supplementary Homicide Reports, and crime laboratory data). NVDRS combines information for each death and links deaths that are related (e.g., multiple homicides, homicide followed by suicide, or multiple suicides) into a single incident. The ability to analyze linked data can provide a more comprehensive understanding of violent deaths. Participating VDRS programs use vital statistics death certificate files or coroner or medical examiner records to identify violent deaths meeting the NVDRS case definition (see Manner of Death). Each VDRS program reports violent deaths of residents that occurred within the state, district, or territory (i.e., resident deaths) and those of nonresidents for whom a fatal injury occurred within the state, district, or territory (i.e., occurrent deaths). When a violent death is identified, NVDRS data abstractors link source documents, link deaths within each incident, code data elements, and write brief narratives of the incident.

In NVDRS, a violent death is defined as a death resulting from the intentional use of physical force or power, threatened or actual, against oneself, another person, or a group or community (*2*). NVDRS collects information on five manners of death: 1) suicide, 2) homicide, 3) legal intervention death, 4) unintentional firearm death, and 5) death of undetermined intent that might have been due to violence (see Manner of Death). NVDRS cases are determined based on *ICD-10* cause of death codes (*5*) or the manner of death assigned by a coroner, medical examiner, or law enforcement officer. Cases are included if they are assigned *ICD-10* cause of death codes (Box 1) or a manner of death specified in at least one of the three primary data sources consistent with NVDRS case definitions.

BOX 1 ***International Classification of Diseases, Tenth Revision*, codes used in the National Violent Death Reporting System, 2019**

**Table:** 

Manner of death	Death ≤1 year after injury	Death >1 year after injury	Death any time after injury
Intentional self-harm (suicide)	X60–X84	Y87.0	U03 (attributable to terrorism)
Assault (homicide)	X85–X99, Y00–Y09	Y87.1	U01, U02 (attributable to terrorism)
Event of undetermined intent	Y10–Y34	Y87.2, Y89.9	Not applicable
Unintentional exposure to inanimate mechanical forces (firearms)	W32–W34	Y86	Not applicable
Legal intervention (excluding executions, Y35.5)	Y35.0–Y35.4, Y35.6, Y35.7	Y89.0	Not applicable

NVDRS is an incident-based system, and all decedents associated with a given incident are grouped in one record. Decisions about whether two or more deaths are related and belong to the same incident are made based on the timing of the injuries rather than on the timing of the deaths. Deaths resulting from injuries that are clearly linked by source documents and occur within 24 hours of one other (see Manner of Death) are considered part of the same incident. Examples of an incident include 1) a single isolated violent death, 2) two or more related homicides (including legal intervention deaths) in which the fatal injuries were inflicted <24 hours apart, 3) two or more related suicides or deaths of undetermined intent in which the fatal injuries were inflicted <24 hours apart, and 4) a homicide followed by a suicide in which both fatal injuries were inflicted <24 hours apart (*6*).

Information collected from each data source is entered into the NVDRS web-based system (*2*). This system streamlines data abstraction by allowing abstractors to enter data from multiple sources into the same incident record. Internal validation checks, hover-over features that define selected fields, and other quality control measures are also included within the system. Primacy rules and hierarchal algorithms related to the source documents occur at the local VDRS program level. CDC provides access to the web-based system to each VDRS program. VDRS program personnel are provided ongoing coding training to learn and adhere to CDC guidance regarding the coding of all variables and technical assistance to help increase data quality. Data are transmitted continuously via the web to a CDC-based server. Information abstracted into the system is deidentified at the local VDRS program level.

### Manner of Death

A manner (i.e., intent) of death for each decedent is assigned by a trained abstractor who integrates information from all source documents. The abstractor-assigned manner of death must be consistent with at least one required data source; typically, all source documents are consistent regarding the manner of death. When a discrepancy exists, the abstractor must assign a manner of death on the basis of a preponderance of evidence in the source documents; however, such occurrences are rare (*6*). For example, if two sources report a death as a suicide and a third reports it as a death of undetermined intent, the death is coded as a suicide.

NVDRS data are categorized into five abstractor-assigned manners of death: 1) suicide, 2) homicide, 3) legal intervention death, 4) unintentional firearm death, and 5) death of undetermined intent. The case definitions for each manner of death are described as follows:

- **Suicide.** A suicide is a death among persons aged ≥10 years resulting from the use of force against oneself when a preponderance of evidence indicates that the use of force was intentional. This category also includes the following scenarios: 1) deaths of persons who intended only to injure rather than die by suicide; 2) persons who initially intended to die by suicide and changed their minds but still died as a result of the act; 3) deaths associated with risk-taking behavior without clear intent to inflict a fatal self-injury but associated with high risk for death (e.g., participating in Russian roulette); 4) suicides that occurred while under the influence of substances taken voluntarily; 5) suicides among decedents with mental health problems that affected their thinking, feelings, or mood (e.g., while experiencing an acute episode of a mental health condition, such as schizophrenia or other psychotic conditions, depression, or posttraumatic stress disorder); and 6) suicides involving another person who provided passive (only) assistance to the decedent (e.g., supplying the means or information needed to complete the act). This category does not include deaths caused by chronic or acute substance use without the intent to die, deaths attributed to autoerotic behavior (e.g., self-strangulation during sexual activity), or assisted suicides (legal or nonlegal). Corresponding *ICD-10* codes included in NVDRS are X60–X84, Y87.0, and U03 (Box 1).
- **Homicide.** A homicide is a death resulting from the use of physical force or power, threatened or actual, against another person, group, or community when a preponderance of evidence indicates that the use of force was intentional. Two special scenarios that CDC’s National Center for Health Statistics (NCHS) regards as homicides are included in the NVDRS case definition: 1) arson with no specified intent to injure someone and 2) a stabbing with intent unspecified. This category also includes the following scenarios: 1) deaths when the suspect intended to only injure rather than kill the victim, 2) deaths resulting from a heart attack induced when the suspect used force or power against the victim, 3) deaths that occurred when a person killed an attacker in self-defense, 4) deaths resulting from a weapon that discharged unintentionally while being used to control or frighten the victim, 5) deaths attributed to child abuse without intent being specified, 6) deaths attributed to an intentional act of neglect by one person against another, 7) deaths of liveborn infants that resulted from a direct injury due to violence sustained before birth, and 8) deaths identified as a justifiable homicide when the person committing homicide was not a law enforcement officer. This category excludes vehicular homicide without intent to injure, unintentional poisoning deaths due to illicit or prescription drug overdose even when the person who provided drugs was charged with homicide, unintentional firearm deaths (a separate category in NVDRS), combat deaths or acts of war, deaths of unborn fetuses, and deaths of infants that resulted indirectly from violence sustained by the mother before birth (e.g., death from prematurity after premature labor brought on by violence). Corresponding *ICD-10* codes included in NVDRS are X85–X99, Y00–Y09, Y87.1, and U01–U02 (Box 1).
- **Legal intervention.** A death from legal intervention is a death in which a person is killed or died as a result of injuries inflicted by a law enforcement officer or another peace officer (i.e., a person with specified legal authority to use deadly force), including military police, while acting in the line of duty. The term “legal intervention” is a classification from *ICD-10* (Y35.0) and does not denote the lawfulness or legality of the circumstances surrounding a death caused by law enforcement. Legal intervention deaths also include a small subset of cases in which force was applied without clear lethal intent (e.g., during restraint or when applying force with a typically nondeadly weapon, such as a Taser) or in which the death occurred while the person was fleeing capture. This category excludes legal executions. Corresponding *ICD-10* codes included in NVDRS are Y35.0–Y35.4, Y35.6, Y35.7, and Y89.0 (Box 1).
- **Unintentional firearm.** An unintentional firearm death is a death resulting from a penetrating injury or gunshot wound from a weapon that uses a powder charge to fire a projectile and for which a preponderance of evidence indicates that the shooting was not directed intentionally at the decedent. Examples include the following: 1) a person who received a self-inflicted wound while playing with a firearm; 2) a person who mistakenly believed a gun was unloaded and shot another person; 3) a child aged <6 years who shot himself or herself or another person; 4) a person who died as a result of a celebratory firing that was not intended to frighten, control, or harm anyone; 5) a person who unintentionally shot himself or herself when using a firearm to frighten, control, or harm another person; 6) a soldier who was shot during a field exercise but not in a combat situation; and 7) an infant who died after birth from an unintentional firearm injury that was sustained in utero. This category excludes injuries caused by unintentionally striking a person with the firearm (e.g., hitting a person on the head with the firearm rather than firing a projectile) and unintentional injuries from nonpowder guns (e.g., BB, pellet, or other compressed-air–powered or compressed-gas–powered guns). Corresponding *ICD-10* codes included in NVDRS are W32–W34 and Y86 (Box 1).
- **Undetermined intent.** A death of undetermined intent is a death resulting from the use of force or power against oneself or another person for which the evidence indicating one manner of death is no more compelling than evidence indicating another. This category includes coroner or medical examiner rulings in which records from data providers indicate that investigators did not find enough evidence to determine whether the injury was intentional (e.g., unclear whether a drug overdose was unintentional or a suicide). Corresponding *ICD-10* codes included in NVDRS are Y10–Y34, Y87.2, and Y89.9 (Box 1).

### Variables Analyzed

NVDRS collects up to approximately 600 unique variables for each death (Boxes 1, 2, and 3). The number of variables recorded for each incident depends on the content and completeness of the source documents. Variables in NVDRS include

BOX 2 **Methods used to inflict injury — National Violent Death Reporting System, 2019**

- Firearm: method that uses a powder charge to fire a projectile from the weapon (excludes BB gun, pellet gun, or compressed air or gas-powered gun)
- Hanging, strangulation, or suffocation (e.g., hanging by the neck, manual strangulation, or plastic bag over the head)
- Poisoning (e.g., fatal ingestion of a street drug, pharmaceutical, carbon monoxide, gas, rat poison, or insecticide)
- Sharp instrument (e.g., knife, razor, machete, or pointed instrument)
- Blunt instrument (e.g., club, bat, rock, or brick)
- Fall: being pushed or jumping
- Motor vehicle (e.g., car, bus, motorcycle, or other transport vehicle)
- Personal weapons (e.g., hands, fists, or feet)
- Drowning: inhalation of liquid (e.g., in bathtub, lake, or other source of water or liquid)
- Fire or burns: inhalation of smoke or the direct effects of fire or chemical burns
- Intentional neglect: starvation, lack of adequate supervision, or withholding of health care
- Other (single method): any method other than those already listed (e.g., electrocution, exposure to environment or weather, or explosives)
- Unknown: method not reported or not known

BOX 3 **Circumstances preceding fatal injury, by manner of death — National Violent Death Reporting System, 2019**

**Suicide or Death of Undetermined Intent**

- Intimate partner problem: decedent was experiencing problems with a current or former intimate partner.
- Suicide of family member or friend: decedent was distraught over, or reacting to, the recent suicide of a family member or friend.
- Other death of family member or friend: decedent was distraught over, or reacting to, the recent non-suicide death of a family member or friend.
- Physical health problem: decedent was experiencing physical health problems (e.g., a recent cancer diagnosis or chronic pain).
- Job problem: decedent was either experiencing a problem at work or was having a problem with joblessness.
- Recent criminal legal problem: decedent was facing criminal legal problems (e.g., recent or impending arrest or upcoming criminal court date).
- Noncriminal legal problem: decedent was facing civil legal problems (e.g., a child custody or civil lawsuit).
- Financial problem: decedent was experiencing financial problems (e.g., bankruptcy, overwhelming debt, or foreclosure of a home or business).
- Eviction or loss of home: decedent was experiencing a recent or impending eviction or other loss of housing, or the threat of eviction or loss of housing.
- School problem: decedent was experiencing a problem related to school (e.g., poor grades, bullying, social exclusion at school, or performance pressures).
- Traumatic anniversary: the incident occurred on or near the anniversary of a traumatic event in the decedent’s life.
- Exposure to disaster: decedent was exposed to a disaster (e.g., earthquake or bombing).
- Left a suicide note: decedent left a note, email message, video, or other communication indicating intent to die by suicide.
- Disclosed suicidal intent: decedent had recently expressed suicidal feelings to another person with time for that person to intervene.
- Disclosed intent to whom: type of person (e.g., family member or current or former intimate partner) to whom the decedent recently disclosed suicidal thoughts or plans.
- History of suicidal thoughts or plans: decedent had previously expressed suicidal thoughts or plans.
- History of attempting suicide: decedent had previously attempted suicide before the fatal incident.

**Homicide or Legal Intervention Death**

- Jealousy (lovers’ triangle): jealousy or distress over an intimate partner’s relationship or suspected relationship with another person.
- Stalking: pattern of unwanted harassing or threatening tactics by either the decedent or suspect.
- Prostitution: prostitution or related activity that includes prostitutes, pimps, clients, or others involved in such activity.
- Drug involvement: drug dealing, drug trade, or illicit drug use that is suspected to have played a role in precipitating the incident.
- Brawl: mutual physical fight involving three or more persons.
- Mercy killing: decedent wished to die because of a terminal or hopeless disease or condition, and documentation indicates that the decedent wanted to be killed.
- Victim was a bystander: decedent was not the intended target in the incident (e.g., pedestrian walking past a gang fight).
- Victim was a police officer on duty: decedent was a law enforcement officer killed in the line of duty.
- Victim was an intervener assisting a crime victim: decedent was attempting to assist a crime victim at the time of the incident (e.g., a child attempts to intervene and is killed while trying to assist a parent who is being assaulted).
- Victim used a weapon: decedent used a weapon to attack or defend during the course of the incident.
- Intimate partner violence related: incident is related to conflict between current or former intimate partners; includes the death of an intimate partner or nonintimate partner (e.g., child, parent, friend, or law enforcement officer) killed in an incident that originated in a conflict between intimate partners.
- Hate crime: decedent was selected intentionally because of his or her actual or perceived gender, religion, sexual orientation, race, ethnicity, or disability.
- Mentally ill suspect: suspect’s attack on decedent was believed to be the direct result of a mental health problem (e.g., schizophrenia or other psychotic condition, depression, or posttraumatic stress disorder).
- Drive-by shooting: suspect drove near the decedent and fired a weapon while driving.
- Walk-by assault: decedent was killed by a targeted attack (e.g., ambush) where the suspect fled on foot.
- Random violence: decedent was killed in a random act of violence (i.e., an act in which the suspect is not concerned with who is being harmed, just that someone is being harmed).
- Gang related: incident resulted from gang activity or gang rivalry; not used if the decedent was a gang member and the death did not appear to result from gang activity.
- Justifiable self-defense: decedent was killed by a law enforcement officer in the line of duty or by a civilian in legitimate self-defense or in defense of others.

**Suspect Information**

- Suspected other substance use by suspect: suspected substance use by the suspect in the hours preceding the incident.
- Suspected alcohol use by suspect: suspected alcohol use by the suspect in the hours preceding the incident.
- Suspect had developmental disability: suspect had developmental disability at time of incident.
- Mentally ill suspect: suspect’s attack on decedent was believed to be the direct result of a mental health problem (e.g., schizophrenia or other psychotic condition, depression, or posttraumatic stress disorder).
- Prior contact with law enforcement: suspect had contact with law enforcement in the past 12 months.
- Suspect attempted suicide after incident: suspect attempted suicide (fatally or non-fatally) after the death of the victim.
- Suspect recently released from an institution: suspect injured victim within a month of being released from or admitted to an institutional setting.
**All Manners of Death (Except Unintentional Firearm)**
- Current depressed mood: decedent was perceived by self or others to be feeling depressed at the time of death.
- Current diagnosed mental health problem: decedent was identified as having a mental health disorder or syndrome listed in the *Diagnostic and Statistical Manual, Version V* (*DSM-V*), with the exception of alcohol and other substance dependence (these are captured in separate variables).
- Type of mental health diagnosis: identifies the type of *DSM-V* diagnosis reported for the decedent.
- Current mental health treatment: decedent was receiving mental health treatment as evidenced by a current prescription for a psychotropic medication, visit or visits to a mental health professional, or participation in a therapy group within the previous 2 months.
- History of ever being treated for mental health problem: decedent was identified as having ever received mental health treatment.
- Alcohol problem: decedent was perceived by self or others to have a problem with, or to be addicted to, alcohol.
- Substance use problem (excludes alcohol): decedent was perceived by self or others to have a problem with, or be addicted to, a substance other than alcohol.
- Other addiction: decedent was perceived by self or others to have an addiction other than to alcohol or other substance (e.g., gambling or sex).
- Family relationship problem: decedent was experiencing problems with a family member, other than an intimate partner.
- Other relationship problem (nonintimate): decedent was experiencing problems with a friend or associate (other than an intimate partner or family member).
- History of child abuse or neglect: as a child, decedent had history of physical, sexual, or psychological abuse; physical (including medical or dental), emotional, or educational neglect; exposure to a violent environment, or inadequate supervision by a caretaker.
- Caretaker abuse or neglect led to death: decedent was experiencing physical, sexual, or psychological abuse; physical (including medical or dental), emotional, or educational neglect; exposure to a violent environment; or inadequate supervision by a caretaker that led to death.
- Perpetrator of interpersonal violence during previous month: decedent perpetrated interpersonal violence during the previous month.
- Victim of interpersonal violence during previous month: decedent was the target of interpersonal violence during the past month.
- Physical fight (two persons, not a brawl): a physical fight between two individuals that resulted in the death of the decedent, who was either involved in the fight, a bystander, or trying to stop the fight.
- Argument or conflict: a specific argument or disagreement led to the victim’s death.
- Precipitated by another crime: incident occurred as the result of another serious crime.
- Nature of crime: the specific type of other crime that occurred during the incident (e.g., robbery or drug trafficking).
- Crime in progress: another serious crime was in progress at the time of the incident.
- Terrorist attack: decedent was injured in a terrorist attack, leading to death.
- Crisis during previous or upcoming 2 weeks: current crisis or acute precipitating event or events that either occurred during the previous 2 weeks or was impending in the following 2 weeks (e.g., a trial for a criminal offense begins the following week) and appeared to have contributed to the death. Crises typically are associated with specific circumstance variables (e.g., job problem was a crisis, or a financial problem was a crisis).
- Other crisis: a crisis related to a death but not captured by any of the standard circumstances.

**Unintentional Firearm Death**

*Context of Injury*

- Hunting: death occurred any time after leaving home for a hunting trip and before returning home from a hunting trip.
- Target shooting: shooter was aiming for a target and unintentionally hit the decedent; can be at a shooting range or an informal backyard setting (e.g., teenagers shooting at signposts on a fence).
- Loading or unloading gun: gun discharged when the shooter was loading or unloading ammunition.
- Cleaning gun: shooter pulled trigger or gun discharged while cleaning, repairing, assembling, or disassembling gun.
- Showing gun to others: gun was being shown to another person when it discharged, or the trigger was pulled.
- Playing with gun: shooter was playing with a gun when it discharged.
- Celebratory firing: shooter fired gun in celebratory manner (e.g., firing into the air at midnight on New Year’s Eve).
- Other context of injury: shooting occurred during some context other than those already described.

*Mechanism of Injury*

- Unintentionally pulled trigger: shooter unintentionally pulled the trigger (e.g., while grabbing the gun or holding it too tightly).
- Thought gun safety was engaged: shooter thought the safety was on and gun would not discharge.
- Thought unloaded or magazine disengaged: shooter thought the gun was unloaded because the magazine was disengaged.
- Thought gun was unloaded: shooter thought the gun was unloaded for other unspecified reason.
- Bullet ricocheted: bullet ricocheted from its intended target and struck the decedent.
- Gun fired due to defect or malfunction: gun had a defect or malfunctioned as determined by a trained firearm examiner.
- Gun fired while holstering: gun was being replaced or removed from holster or clothing.
- Gun was dropped: gun discharged when it was dropped.
- Gun fired while operating safety or lock: shooter unintentionally fired the gun while operating the safety or lock.
- Gun was mistaken for toy: gun was mistaken for a toy and was fired without the user understanding the danger.
- Other mechanism of injury: shooting occurred as the result of a mechanism not already described.

- manner of death (i.e., the intent to cause death [suicide, homicide, legal intervention, unintentional, and undetermined] of the person on whom a fatal injury was inflicted) (Box 1);
- demographic information (e.g., age, sex, and race and ethnicity) of victims and suspects (if applicable);
- method of injury (i.e., the mechanism used to inflict a fatal injury) (Box 2);
- location, date, and time of injury and death;
- toxicology findings (for decedents who were tested);
- circumstances (i.e., the events that preceded and were identified by investigators as relevant and therefore might have contributed to the infliction of a fatal injury) (Box 3);
- whether the decedent was a victim (i.e., a person who died as a result of a violence-related injury) or both a suspect and a victim (i.e., a person believed to have inflicted a fatal injury on a victim who then was fatally injured, such as the perpetrator of a homicide followed by suicide);
- information about any known suspects (i.e., a person or persons believed to have inflicted a fatal injury on a victim);
- incident (i.e., an occurrence in which one or more persons sustained a fatal injury that was linked to a common event or perpetrated by the same suspect or suspects during a 24-hour period); and
- type of incident (i.e., a combination of the manner of death and the number of victims in an incident).

### Circumstances Preceding Death

Circumstances preceding death are defined as the precipitating events that contributed to the infliction of a fatal injury (Box 3). Circumstances are reported based on the content of coroner or medical examiner and law enforcement investigative reports. Certain circumstances are coded to a specific manner of death (e.g., “history of suicidal thoughts or plans” is collected for suicides and deaths of undetermined intent); other circumstances are coded across all manners of death (e.g., “ever treated for mental health or substance use problem”). The data abstractor selects from a list of potential circumstances and is required to code all circumstances that are known to relate to each incident. If circumstances are unknown (e.g., a body found in the woods with no other details reported), the data abstractor does not endorse circumstances; these deaths are then excluded from the denominator for circumstance values. If either the coroner or medical examiner record or law enforcement report indicates the presence of a circumstance, then the abstractor endorses the circumstance. For example, if a law enforcement report indicates that a decedent had disclosed thoughts of suicide or an intent to die by suicide, then the circumstance variable “recent disclosure of suicidal thoughts or intent” is endorsed.

Data abstractors draft two incident narratives: one that summarizes the sequence of events of the incident from the perspective of the coroner or medical examiner record and one that summarizes the sequence of events of the incident from the perspective of the law enforcement report. In addition to briefly summarizing the incident (i.e., the “who, what, when, where, and why” of the incident), the narratives provide supporting information on circumstances that the data abstractor indicated and context for understanding the incident, record information and additional detail that cannot be captured elsewhere, and facilitate data quality control checks on the coding of key variables.

### Coding Training and Quality Control

Ongoing coding support for data abstractors is provided by CDC through an electronic help desk, monthly conference calls, annual in-person or virtual meetings that include coding training for data abstractors, and regular conference calls with individual VDRS programs. In addition, all data abstractors are invited to participate in monthly coding workgroup calls. VDRS programs can conduct additional abstractor training workshops and activities at their own discretion, including through the use of NVDRS Data Abstractor eLearn Training Modules. An NVDRS coding manual (*6*) with CDC-issued standard guidance on coding criteria and examples for each data element is provided to each VDRS program. Software features that enhance coding reliability include automated validation rules and a hover-over feature containing variable-specific information.

Each year, VDRS programs are required to reabstract a subset of cases using multiple abstractors to identify inconsistencies. In addition, each VDRS program’s data quality plan is evaluated by CDC. Before the data are released each year, CDC conducts a quality control analysis that involves the review of multiple variables for data inconsistencies, with special focus on abstractor-assigned variables (e.g., method of injury and manner of death). If CDC finds inconsistencies, the VDRS program is notified and asked for a response or correction. VDRS programs must meet CDC standards for completeness of circumstance data to be included in the national data set. VDRS programs must have circumstance information abstracted from either the coroner or medical examiner record or the law enforcement report for at least 50% of cases. However, VDRS programs often far exceed this requirement. For 2019, a total of 85.7% of suicides, homicides, and legal intervention deaths in NVDRS had circumstance data from either the coroner or medical examiner record or the law enforcement report. In addition, core variables that represent demographic characteristics (e.g., age, sex, and race and ethnicity) and manners of death were missing or unknown for <0.1% of cases. To ensure the final data set has no duplicate records, during the data closeout process, NVDRS first identifies any records within VDRS programs that match on a subset of 14 key variables and then asks VDRS programs to review these records to determine whether they are true duplicates. One record in any set of two or more records that are true duplicates is retained, and the others are deleted by the VDRS program. Next, NVDRS uses SAS (version 9.4; SAS Institute) to search for any instances of duplicates of a unique identification variable associated with each decedent record. As a third and final check for duplicates, the SAS data set is created with an index that only executes successfully if no duplicates of this identification variable are found.

### Time Frame

VDRS programs are required to begin entering all deaths into the web-based system within 4 months from the date the violent death occurred. VDRS programs then have an additional 16 months from the end of the calendar year in which the violent death occurred to complete each incident record. For data collection year 2019 (original completion period through April 2021), VDRS programs were provided an additional 2.5 months (July 15, 2021) to complete incident records because of delays in data collection resulting from the COVID-19 pandemic. Although VDRS programs typically meet timeliness requirements, additional details about an incident occasionally arrive after a deadline has passed. New incidents also might be identified after the deadline (e.g., when a death certificate is revised, new evidence is obtained that changes a manner of death, or an *ICD-10* misclassification is corrected to meet the NVDRS case definition). These additional data are incorporated into NVDRS when analysis files are updated in real time in the web-based system; 2.5 months after the 16-month data collection period for the 2019 data year, case counts increased by <0.1%.

### Inclusion Criteria

The inclusion criteria for violent deaths in this report are as follows: 1) cases met the NVDRS case definition for violent death; 2) cases occurred in participating VDRS states, the District of Columbia, or Puerto Rico in 2019; and 3) at least 50% of cases for each included state, district, or territory had circumstance information collected from the coroner or medical examiner record or law enforcement report. Data for Arkansas, Florida, Idaho, Mississippi, South Dakota, Tennessee, and Texas were ineligible to be included in this report because these states were each in an optional pilot year for data collection year 2019 and their approved and funded data collection plan represented <50% of all violent deaths in their state, or their data were ineligible to be included in this report because the data did not meet the completeness threshold for circumstances. Data collected in 2019 for New York was ineligible to be included in this report because the data did not meet the completeness threshold for circumstances.

Of the participating VDRS programs, 39 states (Alabama, Alaska, Arizona, Colorado, Connecticut, Delaware, Georgia, Hawaii, Indiana, Iowa, Kansas, Kentucky, Louisiana, Maine, Maryland, Massachusetts, Michigan, Minnesota, Missouri, Montana, Nebraska, Nevada, New Hampshire, New Jersey, New Mexico, North Carolina, North Dakota, Ohio, Oklahoma, Oregon, Rhode Island, South Carolina, Utah, Vermont, Virginia, Washington, West Virginia, Wisconsin, and Wyoming) collected information on all violent deaths that occurred in their state in 2019. In addition, data were collected on all violent deaths that occurred in the District of Columbia and Puerto Rico in 2019. Two states, Illinois and Pennsylvania, joined NVDRS with plans to collect data on violent deaths in a subset of counties that represented at least 80% of all violent deaths in their state or in counties where at least 1,800 violent deaths occurred. In 2019, these states reported data on a subset of counties that represented at least 80% of violent deaths in their state. Data were collected for 47 counties in Illinois (Adams, Alexander, Bond, Boone, Brown, Bureau, Champaign, Clay, Cook, DeKalb, Douglas, DuPage, Effingham, Fayette, Fulton, Grundy, Henry, Iroquois, Jackson, Jefferson, Kane, Kankakee, Kendall, Lake, Lasalle, Livingston, Logan, McDonough, McHenry, McLean, Macoupin, Madison, Menard, Peoria, Perry, Piatt, Putnam, Rock Island, St. Clair, Sangamon, Schuyler, Stark, Tazewell, Vermilion, Wayne, Will, and Winn) that represented 90% of the state’s population (*7*). In Pennsylvania, data were collected for 40 counties (Adams, Allegheny, Armstrong, Berks, Blair, Bradford, Bucks, Cameron, Cambria, Carbon, Centre, Chester, Clarion, Clearfield, Clinton, Crawford, Dauphin, Delaware, Erie, Fayette, Forest, Greene, Indiana, Jefferson, Lackawanna, Lancaster, Lehigh, Luzerne, Monroe, Montgomery, Northampton, Philadelphia, Schuylkill, Somerset, Sullivan, Susquehanna, Union, Westmoreland, Wyoming, and York) that represented 83% of the state’s population (*7*). California collected data from death certificates for all violent deaths in the state in 2019 (n = 6,586); data for violent deaths that occurred in 30 counties (Amador, Butte, Colusa, Fresno, Glenn, Humboldt, Imperial, Kern, Kings, Lassen, Lake, Los Angeles, Marin, Modoc, Mono, Orange, Placer, Sacramento, San Benito, San Francisco, San Mateo, Santa Cruz, Shasta, Siskiyou, Solano, Sonoma, Tehama, Trinity, Ventura, and Yolo) also included information from coroner or medical examiner records and law enforcement reports and are included throughout the rest of the report (n = 3,645; 55.3%). These 30 counties represented 57% of California’s population (*7*). Because <100% of violent deaths were reported, data from California, Illinois, and Pennsylvania, are not representative of all violent deaths occurring in these three states.

### Analyses

This report includes data for violent deaths that occurred in 42 states (39 states collecting statewide data, 30 California counties, 47 Illinois counties, and 40 Pennsylvania counties), the District of Columbia, and Puerto Rico in 2019. VDRS program-level data were received by CDC by the extended date of July 15, 2021. All data received by CDC as of July 15, 2021, were consolidated and analyzed. The numbers, percentages, and crude rates are presented in aggregate for all deaths by the abstractor-assigned manner of death. The suicide rate was calculated using denominators among populations aged ≥10 years. The rates for other manners of death used denominators among populations of all ages. The rates for cells with frequency <20 are not reported because of the instability of those rates. Denominators for the rates for the three states that did not collect statewide data (California, Illinois, and Pennsylvania) correspond to the populations of the counties from which data were collected. The rates could not be calculated for certain variables (e.g., circumstances) because denominators were unknown.

Bridged-race 2019 population estimates were used as denominators in the crude rate calculations for the 42 states (39 states collecting statewide data, 30 California counties, 47 Illinois counties, and 40 Pennsylvania counties) and District of Columbia (*8*). For compatible numerators for the rate calculations to be derived, records listing multiple races were recoded to a single race, when possible, using race-bridging methods described by NCHS (https://www.cdc.gov/nchs/nvss/bridged_race.htm) (*9*). The rates specific to race and ethnicity are not available for Puerto Rico because the Census Bureau estimates for Puerto Rico do not include race or Hispanic or Latino (Hispanic) origin (*10*). Data for Puerto Rico were analyzed separately. Population estimates by sex and age were used as denominators in the crude rate calculations for Puerto Rico (*11*).

## Results

### Violent Deaths in 42 States and the District of Columbia

For 2019, a total of 42 NVDRS states (39 states collecting statewide data, 30 California counties, 47 Illinois counties, and 40 Pennsylvania counties) and the District of Columbia collected data on 50,374 incidents involving 51,627 deaths (Supplementary Table S1, https://stacks.cdc.gov/view/cdc/116311). Suicides (n = 33,109; 64.1%) accounted for the highest rate of violent death (16.9 per 100,000 population aged ≥10 years), followed by homicides (n = 12,980; 25.1%) (5.8 per 100,000 population). Deaths of undetermined intent (n = 4,504; 8.7%), legal intervention deaths (n = 699; 1.4%), and unintentional firearm deaths (n = 335; <1.0%) occurred at lower rates (2.0, 0.3, and 0.2 per 100,000 population, respectively). Data for deaths by manner that include statewide counts and the rates for California are available (Supplementary Table S2, https://stacks.cdc.gov/view/cdc/116311).

### Suicides

#### Sex, Age Group, and Race and Ethnicity

For 2019, a total of 42 NVDRS states (39 states collecting statewide data, 30 California counties, 47 Illinois counties, and 40 Pennsylvania counties) and the District of Columbia collected data concerning 32,560 incidents involving 33,109 suicide deaths among persons aged ≥10 years (Supplementary Table S1, https://stacks.cdc.gov/view/cdc/116311). The overall suicide rate was 16.9 per 100,000 population aged ≥10 years (Table 1).

**TABLE 1:** Number, percentage,* and rate^†^ of suicides among persons aged ≥10 years,^§^ by selected demographic characteristics of decedent,^¶^ method used, and location in which injury occurred — National Violent Death Reporting System, 42 states and the District of Columbia,** 2019

Characteristic	Male		Female		Total	
	No. (%)	Rate	No. (%)	Rate	No. (%)	Rate
**Age group (yrs)**						
10–14	245 (<1.0)	3.4	133 (1.9)	1.9	**378 (1.1)**	**2.7**
15–19	1,184 (4.6)	16.3	362 (5.1)	5.2	**1,546 (4.7)**	**10.8**
20–24	2,147 (8.3)	28.5	450 (6.3)	6.3	**2,597 (7.8)**	**17.6**
25–29	2,340 (9.0)	28.9	520 (7.3)	6.7	**2,860 (8.6)**	**18.0**
30–34	2,290 (8.8)	30.0	535 (7.5)	7.2	**2,825 (8.5)**	**18.7**
35–44	4,128 (15.9)	29.4	1,205 (16.9)	8.6	**5,333 (16.1)**	**19.0**
45–54	4,122 (15.9)	30.1	1,514 (21.3)	10.8	**5,636 (17.0)**	**20.3**
55–64	4,345 (16.7)	30.8	1,357 (19.1)	9.0	**5,702 (17.2)**	**19.6**
65–74	2,681 (10.3)	26.6	655 (9.2)	5.7	**3,336 (10.1)**	**15.5**
75–84	1,730 (6.7)	36.8	275 (3.9)	4.6	**2,005 (6.1)**	**18.7**
≥85	780 (3.0)	49.3	107 (1.5)	3.7	**887 (2.7)**	**19.9**
Unknown	2 (<1.0)	—^††^	1 (<1.0)	—	**4 (<1.0)**	**—**
**Race and ethnicity**						
White, non-Hispanic	20,932 (80.5)	32.7	5,694 (80.0)	8.6	**26,626 (80.4)**	**20.5**
Black, non-Hispanic	1,847 (7.1)	15.5	474 (6.7)	3.6	**2,321 (7.0)**	**9.3**
American Indian or Alaska Native, non-Hispanic	427 (1.6)	45.6	148 (2.1)	14.9	**575 (1.7)**	**29.8**
Asian or Pacific Islander	799 (3.1)	13.9	294 (4.1)	4.6	**1,093 (3.3)**	**9.0**
Hispanic^§§^	1,905 (7.3)	14.3	492 (6.9)	3.8	**2,397 (7.2)**	**9.1**
Other	67 (<1.0)	—	9 (<1.0)	—	**77 (<1.0)**	**—**
Unknown	17 (<1.0)	—	3 (<1.0)	—	**20 (<1.0)**	**—**
**Method**						
Firearm	14,355 (55.2)	15.0	2,150 (30.2)	2.2	**16,505 (49.9)**	**8.4**
Hanging, strangulation, or suffocation	7,612 (29.3)	7.9	2,150 (30.2)	2.2	**9,763 (29.5)**	**5.0**
Poisoning	1,982 (7.6)	2.1	2,173 (30.5)	2.2	**4,155 (12.5)**	**2.1**
Fall	589 (2.3)	0.6	214 (3.0)	0.2	**803 (2.4)**	**0.4**
Sharp instrument	553 (2.1)	0.6	109 (1.5)	0.1	**662 (2.0)**	**0.3**
Motor vehicles (e.g., buses, motorcycles, or other transport vehicles)	415 (1.6)	0.4	114 (1.6)	0.1	**529 (1.6)**	**0.3**
Drowning	205 (<h1.0)	0.2	107 (1.5)	0.1	**312 (<1.0)**	**0.2**
Fire or burns	94 (<1.0)	0.1	33 (<1.0)	<0.1	**127 (<1.0)**	**<0.1**
Blunt instrument	36 (<1.0)	<0.1	15 (<1.0)	—	**51 (<1.0)**	**<0.1**
Other (e.g., Taser, electrocution, or nail gun, intentional neglect, or personal weapons)	46 (<1.0)	—	12 (<1.0)	—	**58 (<1.0)**	**—**
Unknown	107 (<1.0)	—	37 (<1.0)	—	**144 (<1.0)**	**—**
**Location of injury**						
House or apartment	18,547 (71.4)	19.4	5,582 (78.5)	5.6	**24,129 (72.9)**	**12.3**
Motor vehicle	1,404 (5.4)	1.5	281 (3.9)	0.3	**1,685 (5.1)**	**0.9**
Natural area	1,250 (4.8)	1.3	224 (3.1)	0.2	**1,475 (4.5)**	**0.8**
Street or highway	724 (2.8)	0.8	121 (1.7)	0.1	**845 (2.6)**	**0.4**
Hotel or motel	553 (2.1)	0.6	205 (2.9)	0.2	**758 (2.3)**	**0.4**
Parking lot, public garage, or public transport	448 (1.7)	0.5	87 (1.2)	<0.1	**535 (1.6)**	**0.3**
Park, playground, or sports or athletic area	429 (1.7)	0.5	60 (<1.0)	<0.1	**489 (1.5)**	**0.3**
Jail or prison	410 (1.6)	0.4	35 (<1.0)	<0.1	**445 (1.3)**	**0.2**
Railroad tracks	223 (<1.0)	0.2	51 (<1.0)	<0.1	**274 (<1.0)**	**0.1**
Bridge	194 (<1.0)	0.2	66 (<1.0)	<0.1	**260 (<1.0)**	**0.1**
Commercial or retail area	216 (<1.0)	0.2	28 (<1.0)	<0.1	**244 (<1.0)**	**0.1**
Supervised residential facility	109 (<1.0)	0.1	52 (<1.0)	<0.1	**161 (<1.0)**	**<0.1**
Other location^¶¶^	922 (3.5)	—	155 (2.2)	—	**1,077 (3.3)**	**—**
Unknown	565 (2.2)	—	167 (2.3)	—	**732 (2.2)**	**—**
**Total**	**25,994 (100)**	**27.1**	**7,114 (100)**	**7.1**	**33,109 (100)**	**16.9**

* Percentages might not total 100% due to rounding.

^†^ Per 100,000 population.

^§^ Suicide is not reported for decedents aged <10 years, as per standard in the suicide prevention literature. Denominators for the suicide rates represent the total population aged ≥10 years.

^¶^ Sex was unknown for one decedent.

** Alabama, Alaska, Arizona, California, Colorado, Connecticut, Delaware, District of Columbia, Georgia, Hawaii, Illinois, Indiana, Iowa, Kansas, Kentucky, Louisiana, Maine, Maryland, Massachusetts, Michigan, Minnesota, Missouri, Montana, Nebraska, Nevada, New Hampshire, New Jersey, New Mexico, North Carolina, North Dakota, Ohio, Oklahoma, Oregon, Pennsylvania, Rhode Island, South Carolina, Utah, Vermont, Virginia, Washington, West Virginia, Wisconsin, and Wyoming. Illinois and Pennsylvania collected data on ≥80% of violent deaths in their state, in accordance with requirements under which these states were funded. Data for Illinois are for violent deaths that occurred in 47 counties (Adams, Alexander, Bond, Boone, Brown, Bureau, Champaign, Clay, Cook, DeKalb, Douglas, DuPage, Effingham, Fayette, Fulton, Grundy, Henry, Iroquois, Jackson, Jefferson, Kane, Kankakee, Kendall, Lake, Lasalle, Livingston, Logan, McDonough, McHenry, McLean, Macoupin, Madison, Menard, Peoria, Perry, Piatt, Putnam, Rock Island, St. Clair, Sangamon, Schuyler, Stark, Tazewell, Vermilion, Wayne, Will, and Winn). Data for Pennsylvania are for violent deaths that occurred in 40 counties (Adams, Allegheny, Armstrong, Berks, Blair, Bradford, Bucks, Cameron, Cambria, Carbon, Centre, Chester, Clarion, Clearfield, Clinton, Crawford, Dauphin, Delaware, Erie, Fayette, Forest, Greene, Indiana, Jefferson, Lackawanna, Lancaster, Lehigh, Luzerne, Monroe, Montgomery, Northampton, Philadelphia, Schuylkill, Somerset, Sullivan, Susquehanna, Union, Westmoreland, Wyoming, and York). Data for California are for violent deaths that occurred in 30 counties (Amador, Butte, Colusa, Fresno, Glenn, Humboldt, Imperial, Kern, Kings, Lassen, Lake, Los Angeles, Marin, Modoc, Mono, Orange, Placer, Sacramento, San Benito, San Francisco, San Mateo, Santa Cruz, Shasta, Siskiyou, Solano, Sonoma, Tehama, Trinity, Ventura, and Yolo). Denominators for the rates for these three states (Illinois, Pennsylvania, and California) represent only the populations of the counties from which the data were collected.

^††^ Rate is not reported when number of decedents is <20 or when characteristic response is “other” or “unknown.”

^§§^ Includes persons of any race.

^¶¶^ Other location includes (in descending order): hospital or medical facility; industrial or construction area; farm; preschool, school, college, or school bus; cemetery, graveyard, or other burial ground; office building; abandoned house, building, or warehouse; synagogue, church, or temple; bar or nightclub; and other unspecified location.

The overall suicide rate for males (27.1 per 100,000 population) was 3.8 times the rate for females (7.1 per 100,000 population) (Table 1). The suicide rate for males ranged from 1.8 to 13.3 times the rate for females across age groups and 3.0 to 4.3 times the rate for females across racial and ethnic groups. Adults aged 45–54 years (20.3 per 100,000 population), ≥85 years (19.9 per 100,000 population), 55–64 years (19.6 per 100,000 population), and 35–44 years (19.0 per 100,000 population) had the highest rates of suicide across age groups. White persons accounted for the majority (80.4%) of suicides; however, AI/AN persons had the highest rate of suicide (29.8 per 100,000 population) among all racial and ethnic groups.

Among male suicide decedents, nearly half (48.5%) were aged 35–64 years (Table 1). Men aged ≥85 years had the highest rate of suicide (49.3 per 100,000 population), followed by men aged 75–84 years (36.8 per 100,000 population) and 55–64 years (30.8 per 100,000 population). AI/AN males had the highest rate of suicide (45.6 per 100,000 population), followed by White males (32.7 per 100,000 population). The rate of suicide for AI/AN males was 3.3 times the rate for males with the lowest rate (i.e., non-Hispanic Asian or Pacific Islander [A/PI]; 13.9 per 100,000 population). The suicide rate was 15.5 per 100,000 population for Black males and 14.3 per 100,000 population for Hispanic males.

Among females, women aged 35–64 years accounted for 57.3% of suicides (Table 1). Women aged 45–54 years had the highest rate of suicide (10.8 per 100,000 population). The suicide rate was highest among AI/AN females (14.9 per 100,000 population), followed by White (8.6 per 100,000 population), A/PI (4.6 per 100,000 population), Hispanic (3.8 per 100,000 population), and Black (3.6 per 100,000 population) females. The suicide rate for AI/AN females was 4.1 times the rate for females with the lowest rates (i.e., Black females).

#### Method and Location of Injury

A firearm was used in half (49.9%; 8.4 per 100,000 population) of suicides, followed by hanging, strangulation, or suffocation (29.5%; 5.0 per 100,000 population) and poisoning (12.5%; 2.1 per 100,000 population) (Table 1). Among males, the most common method of injury was a firearm (55.2%), followed by hanging, strangulation, or suffocation (29.3%). Among females, poisoning (30.5%) was the most common method of injury; hanging, strangulation, or suffocation and a firearm were used in equal proportions (30.2%). Among all suicide decedents, the most common location of suicide was a house or apartment (72.9%), followed by a motor vehicle (5.1%), a natural area (4.5%), a street or highway (2.6%), and a hotel or motel (2.3%).

#### Toxicology Results of Decedent

Toxicology tests for blood alcohol concentration (BAC) were conducted for 53.8% of suicide decedents (Table 2). Among those with positive results for alcohol (40.7%), 63.8% had a BAC ≥0.08 g/dL. Tests for the following substances were conducted for the percentage of decedents indicated in parentheses: amphetamines (43.3%), antidepressants (30.0%), benzodiazepines (42.4%), cannabis (more commonly referred to as marijuana; 38.5%), cocaine (42.7%), and opioids (45.1%). Positive results were found for 14.7% of decedents tested for amphetamines. Among those tested for antidepressants, 32.9% had positive results at the time of death, 21.5% of those tested for benzodiazepines had positive results, 25.0% of those tested for cannabis had positive results, and 6.1% of those tested for cocaine had positive results. Test results for opioids (including illicit and prescription opioids) were positive for 21.1% of decedents tested for these substances. Carbon monoxide was tested for a substantially smaller proportion of decedents (4.4%) but was identified in almost half of those decedents (41.1%).

**TABLE 2:** Number* and percentage of suicide decedents tested for alcohol and drugs whose results were positive,^†^ by toxicology variable — National Violent Death Reporting System, 42 states and the District of Columbia,^§^ 2019

Toxicology variable	Tested	Positive
	No. (%)	No. (%)
Blood alcohol concentration^¶^	17,804 (53.8)	7,254 (40.7)
Alcohol <0.08 g/dL	—	1,962 (27.0)
Alcohol ≥0.08 g/dL	—	4,630 (63.8)
Alcohol positive, level unknown	—	662 (9.1)
Amphetamines	14,331 (43.3)	2,106 (14.7)
Anticonvulsants	7,805 (23.6)	1,289 (16.5)
Antidepressants	9,942 (30.0)	3,273 (32.9)
Antipsychotics	7,521 (22.7)	773 (10.3)
Barbiturates	12,223 (36.9)	254 (2.1)
Benzodiazepines	14,032 (42.4)	3,010 (21.5)
Carbon monoxide	1,451 (4.4)	596 (41.1)
Cocaine	14,125 (42.7)	857 (6.1)
Cannabis**	12,760 (38.5)	3,190 (25.0)
Muscle relaxants	7,715 (23.3)	455 (5.9)
Opioids	14,928 (45.1)	3,153 (21.1)
Other drugs or substances**^††^**	2,419 (7.3)	2,294 (94.8)

* Number of suicide decedents = 33,109.

^†^ Percentage is of decedents tested for toxicology. Denominator for the percentage positive is the percentage tested.

^§^ Alabama, Alaska, Arizona, California, Colorado, Connecticut, Delaware, District of Columbia, Georgia, Hawaii, Illinois, Indiana, Iowa, Kansas, Kentucky, Louisiana, Maine, Maryland, Massachusetts, Michigan, Minnesota, Missouri, Montana, Nebraska, Nevada, New Hampshire, New Jersey, New Mexico, North Carolina, North Dakota, Ohio, Oklahoma, Oregon, Pennsylvania, Rhode Island, South Carolina, Utah, Vermont, Virginia, Washington, West Virginia, Wisconsin, and Wyoming. Illinois and Pennsylvania collected data on ≥80% of violent deaths in their state, in accordance with requirements under which these states were funded. Data for Illinois are for violent deaths that occurred in 47 counties (Adams, Alexander, Bond, Boone, Brown, Bureau, Champaign, Clay, Cook, DeKalb, Douglas, DuPage, Effingham, Fayette, Fulton, Grundy, Henry, Iroquois, Jackson, Jefferson, Kane, Kankakee, Kendall, Lake, Lasalle, Livingston, Logan, McDonough, McHenry, McLean, Macoupin, Madison, Menard, Peoria, Perry, Piatt, Putnam, Rock Island, St. Clair, Sangamon, Schuyler, Stark, Tazewell, Vermilion, Wayne, Will, and Winn). Data for Pennsylvania are for violent deaths that occurred in 40 counties (Adams, Allegheny, Armstrong, Berks, Blair, Bradford, Bucks, Cameron, Cambria, Carbon, Centre, Chester, Clarion, Clearfield, Clinton, Crawford, Dauphin, Delaware, Erie, Fayette, Forest, Greene, Indiana, Jefferson, Lackawanna, Lancaster, Lehigh, Luzerne, Monroe, Montgomery, Northampton, Philadelphia, Schuylkill, Somerset, Sullivan, Susquehanna, Union, Westmoreland, Wyoming, and York). Data for California are for violent deaths that occurred in 30 counties (Amador, Butte, Colusa, Fresno, Glenn, Humboldt, Imperial, Kern, Kings, Lassen, Lake, Los Angeles, Marin, Modoc, Mono, Orange, Placer, Sacramento, San Benito, San Francisco, San Mateo, Santa Cruz, Shasta, Siskiyou, Solano, Sonoma, Tehama, Trinity, Ventura, and Yolo).

^¶^ Blood alcohol concentration of ≥0.08 g/dL is greater than the legal limit in all states and the District of Columbia, and is used as the standard for intoxication.

** More commonly referred to as marijuana.

**^††^** Other drugs or substances indicated whether any results were positive; levels for these drugs or substances are not measured.

#### Precipitating Circumstances

Circumstances were identified in 29,723 (89.8%) of suicides (Table 3). Overall, a mental health problem was the most common circumstance, with approximately half (48.3%) of decedents having had a current diagnosed mental health problem and 33.0% experiencing a depressed mood at the time of death. Among the 14,344 decedents with a current diagnosed mental health problem, depression or dysthymia (75.0%), anxiety disorder (22.3%), and bipolar disorder (14.9%) were the most common diagnoses. Among suicide decedents, 25.4% were receiving mental health treatment at the time of death. Alcohol use problems were reported for 19.2% of suicide decedents, and other substance use problems (unrelated to alcohol) were reported for 17.4% of suicide decedents (Table 3).

**TABLE 3:** Number* and percentage^†^ of suicides among persons aged ≥10 years,^§^ by decedent’s sex and precipitating circumstances — National Violent Death Reporting System, 42 states and the District of Columbia,^¶^ 2019

Precipitating circumstance	Male	Female	Total
	No. (%)	No. (%)	No. (%)
**Mental health problem or substance use**			
Current diagnosed mental health problem**	10,180 (43.9)	4,164 (63.6)	**14,344 (48.3)**
Depression or dysthymia	7,519 (73.9)	3,240 (77.8)	**10,759 (75.0)**
Anxiety disorder	2,079 (20.4)	1,124 (27.0)	**3,203 (22.3)**
Bipolar disorder	1,348 (13.2)	783 (18.8)	**2,131 (14.9)**
Posttraumatic stress disorder	654 (6.4)	197 (4.7)	**851 (5.9)**
Schizophrenia	649 (6.4)	200 (4.8)	**849 (5.9)**
Attention deficit disorder or attention deficit hyperactivity disorder	333 (3.3)	67 (1.6)	**400 (2.8)**
Dementia	70 (<1.0)	25 (<1.0)	**95 (<1.0)**
Obsessive compulsive disorder	47 (<1.0)	16 (<1.0)	**63 (<1.0)**
Autism spectrum	37 (<1.0)	5 (<1.0)	**42 (<1.0)**
Eating disorder	6 (<1.0)	25 (<1.0)	**31 (<1.0)**
Other	594 (5.8)	212 (5.1)	**806 (5.6)**
Unknown	672 (6.6)	255 (6.1)	**927 (6.5)**
History of ever being treated for a mental health problem	6,966 (30.1)	3,125 (47.7)	**10,091 (34.0)**
Current depressed mood	7,625 (32.9)	2,184 (33.3)	**9,809 (33.0)**
Current mental health treatment	5,049 (21.8)	2,488 (38.0)	**7,537 (25.4)**
Alcohol problem	4,587 (19.8)	1,113 (17.0)	**5,700 (19.2)**
Substance use problem (excludes alcohol)	3,887 (16.8)	1,283 (19.6)	**5,170 (17.4)**
Other addiction (e.g., gambling or sex)	226 (<1.0)	52 (<1.0)	**278 (<1.0)**
**Interpersonal factors**			
Intimate partner problem	6,271 (27.1)	1,594 (24.3)	**7,865 (26.5)**
Family relationship problem	1,775 (7.7)	631 (9.6)	**2,406 (8.1)**
Other death of family member or friend	1,331 (5.7)	466 (7.1)	**1,797 (6.0)**
Suicide of family member or friend	548 (2.4)	191 (2.9)	**739 (2.5)**
Perpetrator of interpersonal violence during past month	581 (2.5)	57 (<1.0)	**638 (2.1)**
Other relationship problem (nonintimate)	485 (2.1)	150 (2.3)	**635 (2.1)**
Victim of interpersonal violence during past month	71 (<1.0)	59 (<1.0)	**130 (<1.0)**
**Life stressors**			
Crisis during previous or upcoming 2 weeks	6,459 (27.9)	1,571 (24.0)	**8,030 (27.0)**
Physical health problem	4,939 (21.3)	1,305 (19.9)	**6,244 (21.0)**
Argument or conflict	3,675 (15.9)	1,100 (16.8)	**4,775 (16.1)**
Job problem	2,240 (9.7)	408 (6.2)	**2,648 (8.9)**
Financial problem	1,921 (8.3)	442 (6.7)	**2,363 (8.0)**
Recent criminal legal problem	2,093 (9.0)	223 (3.4)	**2,316 (7.8)**
Noncriminal legal problem	845 (3.6)	226 (3.4)	**1,071 (3.6)**
Eviction or loss of home	805 (3.5)	218 (3.3)	**1,023 (3.4)**
School problem	351 (1.5)	117 (1.8)	**468 (1.6)**
History of child abuse or neglect	218 (<1.0)	160 (2.4)	**378 (1.3)**
Physical fight (two persons, not a brawl)	248 (1.1)	33 (<1.0)	**281 (<1.0)**
Traumatic anniversary	167 (<1.0)	67 (1.0)	**234 (<1.0)**
Exposure to disaster	31 (<1.0)	4 (<1.0)	**35 (<1.0)**
Caretaker abuse or neglect led to suicide	13 (<1.0)	12 (<1.0)	**25 (<1.0)**
**Crime and criminal activity**			
Precipitated by another crime	919 (4.0)	101 (1.5)	**1,020 (3.4)**
Crime in progress^††^	296 (32.2)	26 (25.7)	**322 (31.6)**
**Suicide event**			
History of suicidal thoughts or plans	8,163 (35.2)	2,692 (41.1)	**10,855 (36.5)**
Left a suicide note	7,060 (30.5)	2,549 (38.9)	**9,609 (32.3)**
History of attempting suicide	3,718 (16.0)	2,118 (32.3)	**5,836 (19.6)**
**Suicide disclosure**			
Disclosed suicidal intent	5,450 (23.5)	1,553 (23.7)	**7,003 (23.6)**
Disclosed intent to whom^§§^			
Previous or current intimate partner	2,291 (42.0)	555 (35.7)	**2,846 (40.6)**
Other family member	1,686 (30.9)	531 (34.2)	**2,217 (31.7)**
Friend or colleague	673 (12.3)	219 (14.1)	**892 (12.7)**
Health care worker	208 (3.8)	97 (6.2)	**305 (4.4)**
Neighbor	59 (1.1)	21 (1.4)	**80 (1.1)**
Through social media or other electronic means	38 (<1.0)	13 (<1.0)	**51 (<1.0)**
Other	479 (8.8)	110 (7.1)	**589 (8.4)**
Unknown	353 (6.5)	109 (7.0)	**462 (6.6)**
**Total^¶¶^**	**23,172 (89.1)**	**6,551 (92.1)**	**29,723 (89.8)**

* Includes suicides with one or more precipitating circumstances. More than one circumstance could have been present per decedent.

^†^ Denominator includes suicides with one or more precipitating circumstances. The sums of percentages in columns exceed 100% because more than one circumstance could have been present per decedent.

^§^ Suicide is not reported for decedents aged <10 years, as per standard in the suicide prevention literature.

^¶^ Alabama, Alaska, Arizona, California, Colorado, Connecticut, Delaware, District of Columbia, Georgia, Hawaii, Illinois, Indiana, Iowa, Kansas, Kentucky, Louisiana, Maine, Maryland, Massachusetts, Michigan, Minnesota, Missouri, Montana, Nebraska, Nevada, New Hampshire, New Jersey, New Mexico, North Carolina, North Dakota, Ohio, Oklahoma, Oregon, Pennsylvania, Rhode Island, South Carolina, Utah, Vermont, Virginia, Washington, West Virginia, Wisconsin, and Wyoming. Illinois and Pennsylvania collected data on ≥80% of violent deaths in their state, in accordance with requirements under which these states were funded. Data for Illinois are for violent deaths that occurred in 47 counties (Adams, Alexander, Bond, Boone, Brown, Bureau, Champaign, Clay, Cook, DeKalb, Douglas, DuPage, Effingham, Fayette, Fulton, Grundy, Henry, Iroquois, Jackson, Jefferson, Kane, Kankakee, Kendall, Lake, Lasalle, Livingston, Logan, McDonough, McHenry, McLean, Macoupin, Madison, Menard, Peoria, Perry, Piatt, Putnam, Rock Island, St. Clair, Sangamon, Schuyler, Stark, Tazewell, Vermilion, Wayne, Will, and Winn). Data for Pennsylvania are for violent deaths that occurred in 40 counties (Adams, Allegheny, Armstrong, Berks, Blair, Bradford, Bucks, Cameron, Cambria, Carbon, Centre, Chester, Clarion, Clearfield, Clinton, Crawford, Dauphin, Delaware, Erie, Fayette, Forest, Greene, Indiana, Jefferson, Lackawanna, Lancaster, Lehigh, Luzerne, Monroe, Montgomery, Northampton, Philadelphia, Schuylkill, Somerset, Sullivan, Susquehanna, Union, Westmoreland, Wyoming, and York). Data for California are for violent deaths that occurred in 30 counties (Amador, Butte, Colusa, Fresno, Glenn, Humboldt, Imperial, Kern, Kings, Lassen, Lake, Los Angeles, Marin, Modoc, Mono, Orange, Placer, Sacramento, San Benito, San Francisco, San Mateo, Santa Cruz, Shasta, Siskiyou, Solano, Sonoma, Tehama, Trinity, Ventura, and Yolo).

** Includes decedents with one or more diagnosed current mental health problems; therefore, sums of percentages for the diagnosed conditions exceed 100%. Denominator includes the number of decedents with one or more current diagnosed mental health problems.

^††^ Denominator includes those decedents involved in an incident that was precipitated by another crime.

^§§^ Denominator includes decedents who disclosed intent. The sum of percentages exceed 100% because more than one response could have been present per decedent.

^¶¶^ Circumstances were unknown for 3,386 decedents (2,822 males, 563 females, and one unknown); total number of suicide decedents = 33,109 (25,994 males, 7,114 females, and one unknown).

The most commonly reported interpersonal or life stressor precipitating circumstances for suicide were a recent or impending crisis during the previous or upcoming 2 weeks (27.0%), intimate partner problem (26.5%), physical health problem (21.0%), and argument or conflict (16.1%). Among other circumstances related to the suicide, one third (32.3%) of decedents left a suicide note, 36.5% had a history of suicidal thoughts or plans, 19.6% had a history of attempting suicide, and 23.6% had disclosed suicidal intent to another person. Among those who disclosed intent, the greatest proportion of disclosures were to a previous or current intimate partner (40.6%), followed by a family member other than an intimate partner (31.7%) and friend or colleague (12.7%).

When examining known circumstances by sex, a larger percentage of female decedents (63.6%) had a current diagnosed mental health problem than did male decedents (43.9%) (Table 3). Male and female suicide decedents had similar percentages of depressed mood at the time of their death (32.9% and 33.3%, respectively). A larger percentage of female decedents (38.0%) than male decedents (21.8%) were known to have been receiving mental health treatment at the time of death. Suicide events, including leaving a suicide note, history of suicidal thoughts or plans, and history of attempting suicide, occurred more frequently and at higher rates among females than males.

### Homicides

#### Sex, Age Group, and Race and Ethnicity

For 2019, a total of 42 NVDRS states (39 states collecting statewide data, 30 California counties, 47 Illinois counties, and 40 Pennsylvania counties) and the District of Columbia collected data on 11,331 incidents (Supplementary Table S1, https://stacks.cdc.gov/view/cdc/116311) involving 12,980 homicide deaths. The overall homicide rate was 5.8 per 100,000 population (Table 4).

**TABLE 4:** Number, percentage,* and rate^†^ of homicides, by selected demographic characteristics of decedent, method used, location in which injury occurred, and relationship of victim to suspect^§^ — National Violent Death Reporting System, 42 states and the District of Columbia,^¶^ 2019

Characteristic	Male		Female		Total	
	No. (%)	Rate	No. (%)	Rate	No. (%)	Rate
**Age group (yrs)**						
<1	108 (1.0)	8.3	86 (3.2)	7.0	**194 (1.5)**	**7.7**
1–4	119 (1.2)	2.2	83 (3.1)	1.6	**202 (1.6)**	**1.9**
5–9	62 (<1.0)	0.9	42 (1.6)	0.6	**104 (<1.0)**	**0.8**
10–14	79 (<1.0)	1.1	29 (1.1)	0.4	**108 (<1.0)**	**0.8**
15–19	1,099 (10.6)	15.1	170 (6.4)	2.4	**1,269 (9.8)**	**8.9**
20–24	1,705 (16.5)	22.6	292 (11.0)	4.1	**1,997 (15.4)**	**13.6**
25–29	1,721 (16.7)	21.2	299 (11.3)	3.9	**2,020 (15.6)**	**12.7**
30–34	1,351 (13.1)	17.7	268 (10.1)	3.6	**1,619 (12.5)**	**10.7**
35–44	1,833 (17.7)	13.1	457 (17.2)	3.3	**2,290 (17.6)**	**8.2**
45–54	1,042 (10.1)	7.6	343 (12.9)	2.4	**1,385 (10.7)**	**5.0**
55–64	780 (7.6)	5.5	270 (10.2)	1.8	**1,050 (8.1)**	**3.6**
65–74	300 (2.9)	3.0	165 (6.2)	1.4	**465 (3.6)**	**2.2**
75–84	95 (<1.0)	2.0	95 (3.6)	1.6	**190 (1.5)**	**1.8**
≥85	33 (<1.0)	2.1	52 (2.0)	1.8	**85 (<1.0)**	**1.9**
Unknown	1 (<1.0)	—**	0 (0.0)	—	**2 (<1.0)**	**—**
**Race and ethnicity**						
White, non-Hispanic	2,274 (22.0)	3.2	1,209 (45.6)	1.6	**3,483 (26.8)**	**2.4**
Black, non-Hispanic	6,224 (60.3)	44.5	978 (36.9)	6.4	**7,202 (55.5)**	**24.6**
American Indian or Alaska Native, non-Hispanic	196 (1.9)	17.7	80 (3.0)	6.9	**276 (2.1)**	**12.2**
Asian or Pacific Islander	153 (1.5)	2.3	77 (2.9)	1.1	**230 (1.8)**	**1.7**
Hispanic^††^	1,440 (13.9)	8.9	295 (11.1)	1.9	**1,735 (13.4)**	**5.4**
Other	34 (<1.0)	—	9 (<1.0)	—	**44 (<1.0)**	**—**
Unknown	7 (<1.0)	—	3 (<1.0)	—	**10 (<1.0)**	**—**
**Method**						
Firearm	8,118 (78.6)	7.4	1,561 (58.9)	1.4	**9,679 (74.6)**	**4.4**
Sharp instrument	914 (8.8)	0.8	399 (15.1)	0.4	**1,313 (10.1)**	**0.6**
Blunt instrument	367 (3.6)	0.3	169 (6.4)	0.2	**536 (4.1)**	**0.2**
Personal weapons (e.g., hands, feet, or fists)	358 (3.5)	0.3	125 (4.7)	0.1	**483 (3.7)**	**0.2**
Hanging, strangulation, or suffocation	119 (1.2)	0.1	153 (5.8)	0.1	**272 (2.1)**	**0.1**
Motor vehicles (e.g., buses, motorcycles, or other transport vehicles)	69 (<1.0)	<0.1	36 (1.4)	<0.1	**105 (<1.0)**	**<0.1**
Fire or burns	44 (<1.0)	<0.1	27 (1.0)	<0.1	**71 (<1.0)**	**<0.1**
Poisoning	35 (<1.0)	<0.1	25 (<1.0)	<0.1	**60 (<1.0)**	**<0.1**
Shaking (e.g., shaken baby syndrome)	25 (<1.0)	<0.1	17 (<1.0)	—	**42 (<1.0)**	**<0.1**
Intentional neglect	16 (<1.0)	—	25 (<1.0)	<0.1	**41 (<1.0)**	**<0.1**
Fall	21 (<1.0)	<0.1	10 (<1.0)	—	**31 (<1.0)**	**<0.1**
Drowning	11 (<1.0)	—	14 (<1.0)	—	**25 (<1.0)**	**<0.1**
Other (e.g., Taser, electrocution, or nail gun)	10 (<1.0)	—	6 (<1.0)	—	**16 (<1.0)**	**—**
Unknown	221 (2.1)	—	84 (3.2)	—	**306 (2.4)**	**—**
**Location of injury**						
House or apartment	4,082 (39.5)	3.7	1,705 (64.3)	1.5	**5,787 (44.6)**	**2.6**
Street or highway	2,595 (25.1)	2.4	226 (8.5)	0.2	**2,821 (21.7)**	**1.3**
Motor vehicle	1,092 (10.6)	1.0	233 (8.8)	0.2	**1,325 (10.2)**	**0.6**
Parking lot, public garage, or public transport	503 (4.9)	0.5	54 (2.0)	<0.1	**557 (4.3)**	**0.3**
Commercial or retail area	366 (3.5)	0.3	46 (1.7)	<0.1	**412 (3.2)**	**0.2**
Natural area	146 (1.4)	0.1	59 (2.2)	<0.1	**205 (1.6)**	**<0.1**
Bar or nightclub	184 (1.8)	0.2	18 (<1.0)	—	**202 (1.6)**	**<0.1**
Park, playground, or sports or athletic area	138 (1.3)	0.1	27 (1.0)	<0.1	**165 (1.3)**	**<0.1**
Hotel or motel	112 (1.1)	0.1	34 (1.3)	<0.1	**146 (1.1)**	**<0.1**
Other location^§§^	453 (4.4)	—	115 (4.3)	—	**568 (4.4)**	**—**
Unknown	657 (6.4)	—	134 (5.1)	—	**792 (6.1)**	**—**
**Relationship of victim to suspect^¶¶^**						
Acquaintance or friend	1,307 (33.8)	1.2	201 (11.3)	0.2	**1,508 (26.7)**	**0.7**
Spouse or intimate partner (current or former)	278 (7.2)	0.3	906 (50.8)	0.8	**1,184 (21.0)**	**0.5**
Other person, known to victim	761 (19.7)	0.7	143 (8.0)	0.1	**904 (16.0)**	**0.4**
Stranger	669 (17.3)	0.6	95 (5.3)	<0.1	**764 (13.5)**	**0.3**
Other relative	281 (7.3)	0.3	112 (6.3)	0.1	**393 (7.0)**	**0.2**
Child***	208 (5.4)	0.2	145 (8.1)	0.1	**353 (6.2)**	**0.2**
Parent***	188 (4.9)	0.2	146 (8.2)	0.1	**334 (5.9)**	**0.2**
Rival gang member	82 (2.1)	<0.1	1 (<1.0)	—	**83 (1.5)**	**<0.1**
Other relationship^†††^	93 (2.4)	—	34 (1.9)	—	**127 (2.2)**	**—**
**Total**	**10,328 (100)**	**9.4**	**2,651 (100)**	**2.4**	**12,980 (100)**	**5.8**

* Percentages might not total 100% due to rounding. Sex was unknown for one decedent.

^†^ Per 100,000 population.

^§^ The following sentence can be used as a guide for interpreting the relationship between the victim and suspect: “The victim is the [insert relationship] of the suspect.” For example, when a parent kills a child, the relationship is “child” not “parent” (“The victim is the child of the suspect”). Please note that this sentence is intended to be a general guide. However, some relationships might not be captured by this sentence (e.g., other person known to victim; victim was law enforcement officer killed in the line of duty).

^¶^ Alabama, Alaska, Arizona, California, Colorado, Connecticut, Delaware, District of Columbia, Georgia, Hawaii, Illinois, Indiana, Iowa, Kansas, Kentucky, Louisiana, Maine, Maryland, Massachusetts, Michigan, Minnesota, Missouri, Montana, Nebraska, Nevada, New Hampshire, New Jersey, New Mexico, North Carolina, North Dakota, Ohio, Oklahoma, Oregon, Pennsylvania, Rhode Island, South Carolina, Utah, Vermont, Virginia, Washington, West Virginia, Wisconsin, and Wyoming. Illinois and Pennsylvania collected data on ≥80% of violent deaths in their state, in accordance with requirements under which these states were funded. Data for Illinois are for violent deaths that occurred in 47 counties (Adams, Alexander, Bond, Boone, Brown, Bureau, Champaign, Clay, Cook, DeKalb, Douglas, DuPage, Effingham, Fayette, Fulton, Grundy, Henry, Iroquois, Jackson, Jefferson, Kane, Kankakee, Kendall, Lake, Lasalle, Livingston, Logan, McDonough, McHenry, McLean, Macoupin, Madison, Menard, Peoria, Perry, Piatt, Putnam, Rock Island, St. Clair, Sangamon, Schuyler, Stark, Tazewell, Vermilion, Wayne, Will, and Winn). Data for Pennsylvania are for violent deaths that occurred in 40 counties (Adams, Allegheny, Armstrong, Berks, Blair, Bradford, Bucks, Cameron, Cambria, Carbon, Centre, Chester, Clarion, Clearfield, Clinton, Crawford, Dauphin, Delaware, Erie, Fayette, Forest, Greene, Indiana, Jefferson, Lackawanna, Lancaster, Lehigh, Luzerne, Monroe, Montgomery, Northampton, Philadelphia, Schuylkill, Somerset, Sullivan, Susquehanna, Union, Westmoreland, Wyoming, and York). Data for California are for violent deaths that occurred in 30 counties (Amador, Butte, Colusa, Fresno, Glenn, Humboldt, Imperial, Kern, Kings, Lassen, Lake, Los Angeles, Marin, Modoc, Mono, Orange, Placer, Sacramento, San Benito, San Francisco, San Mateo, Santa Cruz, Shasta, Siskiyou, Solano, Sonoma, Tehama, Trinity, Ventura, and Yolo). Denominators for the rates for these three states (Illinois, Pennsylvania, and California) represent only the populations of the counties from which the data were collected.

** Rates are not reported when number of decedents is <20 or when characteristic response is “other” or “unknown.”

^††^ Includes persons of any race.

^§§^ Other location includes (in descending order): jail or prison; abandoned house, building, or warehouse; supervised residential facility; hospital or medical facility; office building; industrial or construction area; preschool, school, college, or school bus; railroad tracks; synagogue, church, or temple; farm; cemetery, graveyard, or other burial ground; bridge; and other unspecified location.

^¶¶^ Percentage is based on the number of homicide decedents with a known victim-to-suspect relationship (n = 5,650 [43.5%]; 3,867 [37.4%] males and 1,783 [67.3%] females); victim-to-suspect relationship was unknown for 7,330 decedents.

*** Includes adoptive family members (e.g., adopted child), stepfamily members (e.g., stepparent), and foster family members (e.g., foster child).

^†††^ Other relationship includes (in descending order): child of suspect’s boyfriend or girlfriend (e.g., child killed by mother’s boyfriend), and intimate partner of suspect’s parent (e.g., teenager kills his mother’s boyfriend), victim was law enforcement officer injured in line of duty, victim was injured by a law enforcement officer.

The homicide rates were higher among males than females across all age groups, and the rate was highest among adults aged 20–24 years (13.6 per 100,000 population) (Table 4). The homicide rate for men aged 20–24 years was 5.5 times the rate for women in the same age group. Among males, the rate of homicide was highest among adults aged 20–24 years (22.6 per 100,000 population) and 25–29 years (21.2 per 100,000 population). Among females, the rate of homicide was highest among children aged <1 year (7.0 per 100,000 population). Among all children who were homicide victims, the overall homicide rate for infants (i.e., children aged <1 year; 7.7 per 100,000 population) was 4.1 times the overall rate for children aged 1–4 years (1.9 per 100,000 population) and 9.6 times the rate for children aged 5–9 years and 10–14 years (both 0.8 per 100,000 population).

Black persons accounted for 60.3% of male homicide victims and more than half (55.5%) of all homicide victims (Table 4). Black males had the highest rate of homicide compared with males in all other racial and ethnic groups (44.5 per 100,000 population); this rate was 19.3 times the rate for A/PI males (2.3 per 100,000 population), 13.9 times the rate for White males (3.2 per 100,000 population), 5.0 times the rate for Hispanic males (8.9 per 100,000 population), and 2.5 times the rate for AI/AN males (17.7 per 100,000 population). Among females, the homicide rate was highest among AI/AN females (6.9 per 100,000 population) (Table 4), followed by Black females (6.4 per 100,000 population), Hispanic females (1.9 per 100,000 population), White females (1.6 per 100,000 population), and A/PI females (1.1 per 100,000 population).

#### Method, Location of Injury, and Victim-Suspect Relationship

A firearm was used in 74.6% of homicides overall, followed by a sharp instrument (10.1%), a blunt instrument (4.1%), personal weapons (e.g., hands, feet, or fists; 3.7%), and hanging, strangulation, or suffocation (2.1%) (Table 4). The method was unknown in 2.4% of homicides. A firearm was the most common method of injury for both males (78.6%) and females (58.9%); however, the firearm homicide rate for males (7.4 per 100,000 population) was 5.3 times the rate for females (1.4 per 100,000 population). A larger proportion of homicides among females than males involved a sharp instrument (15.1% versus 8.8%, respectively); blunt instrument (6.4% versus 3.6%, respectively); hanging, strangulation, or suffocation (5.8% versus 1.2%, respectively); and personal weapons (4.7% versus 3.5%, respectively). Among all homicide victims, a house or apartment was the most common location of homicide (44.6%), followed by a street or highway (21.7%), a motor vehicle (10.2%), and a parking lot, public garage, or public transport (4.3%). A larger proportion of homicides among females (64.3%) than among males (39.5%) occurred at a house or apartment, whereas a larger proportion of homicides among males (25.1%) than among females (8.5%) occurred on a street or highway.

The relationship of the victim to the suspect was known for 43.5% of homicides (37.4% of males and 67.3% of females) (Table 4). For males, when the relationship was known, the victim-suspect relationship was most often an acquaintance or friend (33.8%); other person known to the victim, but the exact nature of the relationship was unclear (19.7%); a stranger (17.3%); other relative (7.3%); or a current or former intimate partner (7.2%). For females, when the relationship was known, half (50.8%) of suspects were a current or former intimate partner, followed by an acquaintance or friend (11.3%); parent (8.2%); child (8.1%); other person known to victim, but the exact nature of the relationship was unclear (8.0%); other relative (6.3%); or stranger (5.3%).

#### Precipitating Circumstances

Precipitating circumstances were identified in 74.8% of homicides (Table 5). One third of homicides with known circumstances were precipitated by an argument or conflict (34.7%). Almost one fifth (16.5%) of homicides with known circumstances were related to intimate partner violence (Table 5). Intimate partner violence–related deaths include deaths related to conflict or violence between current or former intimate partners and also include deaths associated with intimate partner violence that are not deaths of the intimate partners themselves (e.g., a former boyfriend killing an ex-partner’s new boyfriend). Homicides also were commonly precipitated by another crime (24.5%); in 63.3% of those cases, the crime was in progress at the time of the incident. The most frequent types of precipitating crimes were assault or homicide (40.1%), robbery (36.1%), drug trade** (13.3%), burglary (13.1%), motor vehicle theft (4.7%), rape or sexual assault (3.2%), and arson (1.7%) (Supplementary Table S10, https://stacks.cdc.gov/view/cdc/116311). A physical fight between two persons (15.1%) and drug involvement (i.e., drug dealing, drug trade, or drug use; 11.1%) were other common precipitating circumstances.

**TABLE 5:** Number* and percentage^†^ of homicides, by decedent’s sex and precipitating circumstances — National Violent Death Reporting System, 42 states and the District of Columbia,^§^ 2019

Precipitating circumstance	Male	Female	Total
	No. (%)	No. (%)	No. (%)
**Mental health problems and substance use**			
Substance use problem (excludes alcohol)	1,012 (13.4)	240 (11.0)	**1,252 (12.9)**
Current diagnosed mental health problem	299 (4.0)	177 (8.1)	**476 (4.9)**
Alcohol problem	356 (4.7)	87 (4.0)	**443 (4.6)**
History of ever being treated for a mental health problem	176 (2.3)	109 (5.0)	**285 (2.9)**
Current mental health treatment	107 (1.4)	80 (3.7)	**187 (1.9)**
Current depressed mood	31 (<1.0)	24 (1.1)	**55 (<1.0)**
Other addiction (e.g., gambling or sex)	29 (<1.0)	6 (<1.0)	**35 (<1.0)**
**Interpersonal factors**			
Intimate partner violence related	622 (8.3)	985 (45.3)	**1,607 (16.5)**
Other relationship problem (nonintimate)	557 (7.4)	93 (4.3)	**650 (6.7)**
Family relationship problem	408 (5.4)	201 (9.2)	**609 (6.3)**
Jealousy (lovers’ triangle)	201 (2.7)	90 (4.1)	**291 (3.0)**
Victim of interpersonal violence during past month	87 (1.2)	130 (6.0)	**217 (2.2)**
Perpetrator of interpersonal violence during past month	144 (1.9)	14 (<1.0)	**158 (1.6)**
**Life stressors**			
Argument or conflict	2,687 (35.6)	682 (31.4)	**3,369 (34.7)**
Physical fight (two persons, not a brawl)	1,262 (16.7)	203 (9.3)	**1,465 (15.1)**
Crisis during previous or upcoming 2 weeks	339 (4.5)	175 (8.0)	**514 (5.3)**
History of child abuse or neglect	69 (<1.0)	46 (2.1)	**115 (1.2)**
**Crime and criminal activity**			
Precipitated by another crime	1,969 (26.1)	415 (19.1)	**2,384 (24.5)**
Crime in progress^¶^	1,253 (63.6)	255 (61.4)	**1,508 (63.3)**
Drug involvement	957 (12.7)	123 (5.7)	**1,080 (11.1)**
Gang related	876 (11.6)	64 (2.9)	**940 (9.7)**
**Homicide circumstance**			
Drive-by shooting	895 (11.9)	103 (4.7)	**998 (10.3)**
Victim used a weapon	643 (8.5)	30 (1.4)	**673 (6.9)**
Walk-by assault	506 (6.7)	44 (2.0)	**550 (5.7)**
Caretaker abuse or neglect led to death	222 (2.9)	201 (9.2)	**423 (4.4)**
Mentally ill suspect	186 (2.5)	160 (7.4)	**346 (3.6)**
Random violence	248 (3.3)	59 (2.7)	**307 (3.2)**
Justifiable self-defense	291 (3.9)	11 (<1.0)	**302 (3.1)**
Brawl	217 (2.9)	25 (1.1)	**242 (2.5)**
Victim was a bystander	121 (1.6)	71 (3.3)	**192 (2.0)**
Victim was an intervener assisting a crime victim	76 (1.0)	16 (<1.0)	**92 (<1.0)**
Stalking	18 (<1.0)	47 (2.2)	**65 (<1.0)**
Prostitution	21 (<1.0)	29 (1.3)	**50 (<1.0)**
Victim was a police officer on duty	23 (<1.0)	1 (<1.0)	**24 (<1.0)**
Mercy killing	4 (<1.0)	12 (<1.0)	**16 (<1.0)**
Hate crime	9 (<1.0)	1 (<1.0)	**10 (<1.0)**
Total**	**7,539 (73.0)**	**2,175 (82.0)**	**9,714 (74.8)**

* Includes homicides with one or more precipitating circumstances. Total numbers do not equal the sums of the columns because more than one circumstance could have been present per decedent.

^†^ Denominator includes those homicides with one or more precipitating circumstances. The sums of percentages in columns exceed 100% because more than one circumstance could have been present per decedent.

^§^ Alabama, Alaska, Arizona, California, Colorado, Connecticut, Delaware, District of Columbia, Georgia, Hawaii, Illinois, Indiana, Iowa, Kansas, Kentucky, Louisiana, Maine, Maryland, Massachusetts, Michigan, Minnesota, Missouri, Montana, Nebraska, Nevada, New Hampshire, New Jersey, New Mexico, North Carolina, North Dakota, Ohio, Oklahoma, Oregon, Pennsylvania, Rhode Island, South Carolina, Utah, Vermont, Virginia, Washington, West Virginia, Wisconsin, and Wyoming. Illinois and Pennsylvania collected data on ≥80% of violent deaths in their state, in accordance with requirements under which these states were funded. Data for Illinois are for violent deaths that occurred in 47 counties (Adams, Alexander, Bond, Boone, Brown, Bureau, Champaign, Clay, Cook, DeKalb, Douglas, DuPage, Effingham, Fayette, Fulton, Grundy, Henry, Iroquois, Jackson, Jefferson, Kane, Kankakee, Kendall, Lake, Lasalle, Livingston, Logan, McDonough, McHenry, McLean, Macoupin, Madison, Menard, Peoria, Perry, Piatt, Putnam, Rock Island, St. Clair, Sangamon, Schuyler, Stark, Tazewell, Vermilion, Wayne, Will, and Winn). Data for Pennsylvania are for violent deaths that occurred in 40 counties (Adams, Allegheny, Armstrong, Berks, Blair, Bradford, Bucks, Cameron, Cambria, Carbon, Centre, Chester, Clarion, Clearfield, Clinton, Crawford, Dauphin, Delaware, Erie, Fayette, Forest, Greene, Indiana, Jefferson, Lackawanna, Lancaster, Lehigh, Luzerne, Monroe, Montgomery, Northampton, Philadelphia, Schuylkill, Somerset, Sullivan, Susquehanna, Union, Westmoreland, Wyoming, and York). Data for California are for violent deaths that occurred in 30 counties (Amador, Butte, Colusa, Fresno, Glenn, Humboldt, Imperial, Kern, Kings, Lassen, Lake, Los Angeles, Marin, Modoc, Mono, Orange, Placer, Sacramento, San Benito, San Francisco, San Mateo, Santa Cruz, Shasta, Siskiyou, Solano, Sonoma, Tehama, Trinity, Ventura, and Yolo).

^¶^ Denominator includes those decedents involved in an incident that was precipitated by another crime.

** Circumstances were unknown for 3,266 (2,789 males and 476 females); total number of homicide decedents = 12,980 (10,328 males and 2,651 females).

Among the identified homicide circumstances, several differences were noted by decedent’s sex, and intimate partner violence accounted for the largest percentage difference. Intimate partner violence was a precipitating circumstance for approximately 45.3% of homicides among females but only 8.3% of homicides among males (Table 5). A larger proportion of female homicide victims than male homicide victims resulted from caregiver abuse or neglect (9.2% versus 2.9%) or were perpetrated by a suspect with a mental health problem (e.g., schizophrenia or other psychotic conditions, depression, or posttraumatic stress disorder; 7.4% versus 2.5%). A larger proportion of male homicide victims than female homicide victims were preceded by a physical fight (16.7% versus 9.3%, respectively), involved drugs (12.7% versus 5.7%, respectively), or were gang related (11.6% versus 2.9%, respectively). A larger proportion of male homicide victims (8.5%) than female homicide victims (1.4%) also were reported to have used a weapon during the incident.

### Legal Intervention Deaths

#### Sex, Age Group, and Race and Ethnicity

For 2019, a total of 42 NVDRS states (39 states collecting statewide data, 30 California counties, 47 Illinois counties, and 40 Pennsylvania counties) and the District of Columbia collected data on 690 incidents involving 699 legal intervention deaths (Supplementary Table S1, https://stacks.cdc.gov/view/cdc/116311). The highest rate of legal intervention death by age group was among men aged 25–29 years (1.5 per 100,000 population), followed by men aged 30–34 years (1.3 per 100,000 population) and 35–44 years (1.2 per 100,000 population) (Table 6). Almost all legal intervention deaths were among males (94.6%). Although White males accounted for nearly half (44.1%) of all legal intervention deaths, AI/AN males had the highest legal intervention death rate (2.4 per 100,000 population), representing a rate 6 times that of White males (0.4 per 100,000 population). The legal intervention death rate for Black males (1.4 per 100,000 population) was 3.5 times the rate for White males. The legal intervention death rate for Hispanic males was 0.8 per 100,000 population.

**TABLE 6:** Number, percentage,* and rate^†^ of legal intervention^§^ deaths, by selected demographic characteristics of decedent, method used, and location in which injury occurred — National Violent Death Reporting System, 42 states and the District of Columbia,^¶^ 2019

Characteristic	Male		Female		Total	
	No. (%)	Rate	No. (%)	Rate	No. (%)	Rate
**Age group (yrs)**						
<10	0 (0.0)	—**	0 (0.0)	—	**0 (0.0)**	**—**
10–14	1 (<1.0)	—	0 (0.0)	—	**1 (<1.0)**	**—**
15–19	28 (4.2)	0.4	2 (7.1)	—	**30 (4.3)**	**0.2**
20–24	66 (9.8)	0.9	3 (10.7)	—	**69 (9.9)**	**0.5**
25–29	122 (18.2)	1.5	3 (10.7)	—	**125 (17.9)**	**0.8**
30–34	101 (15.1)	1.3	4 (14.3)	—	**105 (15.0)**	**0.7**
35–44	164 (24.4)	1.2	5 (17.9)	—	**169 (24.2)**	**0.6**
45–54	110 (16.4)	0.8	8 (28.6)	—	**118 (16.9)**	**0.4**
55–64	63 (9.4)	0.5	2 (7.1)	—	**65 (9.3)**	**0.2**
65–74	9 (1.3)	—	1 (3.6)	—	**10 (1.4)**	**—**
75–84	7 (1.0)	—	0 (0.0)	—	**7 (1.0)**	**—**
≥85	0 (0.0)	—	0 (0.0)	—	**0 (0.0)**	**—**
**Race and ethnicity**						
White, non-Hispanic	296 (44.1)	0.4	13 (46.4)	—	**309 (44.2)**	**0.2**
Black, non-Hispanic	198 (29.5)	1.4	6 (21.4)	—	**204 (29.2)**	**0.7**
American Indian or Alaska Native, non-Hispanic	26 (3.9)	2.4	1 (3.6)	—	**27 (3.9)**	**1.2**
Asian or Pacific Islander	20 (3.0)	0.3	1 (3.6)	—	**21 (3.0)**	**0.2**
Hispanic^††^	129 (19.2)	0.8	6 (21.4)	—	**135 (19.3)**	**0.4**
Other	2 (<1.0)	—	1 (3.6)	—	**3 (<1.0)**	**—**
**Method**						
Firearm	595 (88.7)	0.5	20 (71.4)	<0.1	**615 (88.0)**	**0.3**
Motor vehicles (e.g., buses, motorcycles, or other transport vehicles)	28 (4.2)	<0.1	4 (14.3)	—	**32 (4.6)**	**<0.1**
Personal weapons (e.g., hands, feet, or fists)	10 (1.5)	—	0 (0.0)	—	**10 (1.4)**	**—**
Hanging, strangulation, or suffocation	6 (<1.0)	—	0 (0.0)	—	**6 (<1.0)**	**—**
Fall	5 (<1.0)	—	0 (0.0)	—	**5 (<1.0)**	**—**
Blunt instrument	3 (<1.0)	—	1 (3.6)	—	**4 (<1.0)**	**—**
Drowning	4 (<1.0)	—	0 (0.0)	—	**4 (<1.0)**	**—**
Poisoning	1 (<1.0)	—	1 (3.6)	—	**2 (<1.0)**	**—**
Sharp instrument	1 (<1.0)	—	0 (0.0)	—	**1 (<1.0)**	**—**
Other (e.g., Taser, electrocution, or nail gun)	5 (<1.0)	—	0 (0.0)	—	**5 (<1.0)**	**—**
Unknown	13 (1.9)	—	2 (7.1)	—	**15 (2.1)**	**—**
**Location of injury**						
House or apartment	237 (35.3)	0.2	11 (39.3)	—	**248 (35.5)**	**0.1**
Street or highway	171 (25.5)	0.2	4 (14.3)	—	**175 (25.0)**	**<0.1**
Motor vehicle	69 (10.3)	<0.1	5 (17.9)	—	**74 (10.6)**	**<0.1**
Parking lot, public garage, or public transport	50 (7.5)	<0.1	0 (0.0)	—	**50 (7.2)**	**<0.1**
Commercial or retail area	42 (6.3)	<0.1	2 (7.1)	—	**44 (6.3)**	**<0.1**
Natural area	17 (2.5)	—	0 (0.0)	—	**17 (2.4)**	**—**
Jail or prison	9 (1.3)	—	0 (0.0)	—	**9 (1.3)**	**—**
Office building	7 (1.0)	—	0 (0.0)	—	**7 (1.0)**	**—**
Hotel or motel	5 (<1.0)	—	1 (3.6)	—	**6 (<1.0)**	**—**
Park, playground, or sports or athletic area	5 (<1.0)	—	0 (0.0)	—	**5 (<1.0)**	**—**
Other location^§§^	34 (5.1)	—	1 (3.6)	—	**35 (5.0)**	**—**
Unknown	25 (3.7)	—	4 (14.3)	—	**29 (4.1)**	**—**
**Total**	**671 (100)**	**0.6**	**28 (100)**	**<0.1**	**699 (100)**	**0.3**

* Percentages might not total 100% due to rounding.

^†^ Per 100,000 population.

^§^ The term “legal intervention” does not denote the lawfulness or legality of the circumstances surrounding the death.

^¶^ Alabama, Alaska, Arizona, California, Colorado, Connecticut, Delaware, District of Columbia, Georgia, Hawaii, Illinois, Indiana, Iowa, Kansas, Kentucky, Louisiana, Maine, Maryland, Massachusetts, Michigan, Minnesota, Missouri, Montana, Nebraska, Nevada, New Hampshire, New Jersey, New Mexico, North Carolina, North Dakota, Ohio, Oklahoma, Oregon, Pennsylvania, Rhode Island, South Carolina, Utah, Vermont, Virginia, Washington, West Virginia, Wisconsin, and Wyoming. Illinois and Pennsylvania collected data on ≥80% of violent deaths in their state, in accordance with requirements under which these states were funded. Data for Illinois are for violent deaths that occurred in 47 counties (Adams, Alexander, Bond, Boone, Brown, Bureau, Champaign, Clay, Cook, DeKalb, Douglas, DuPage, Effingham, Fayette, Fulton, Grundy, Henry, Iroquois, Jackson, Jefferson, Kane, Kankakee, Kendall, Lake, Lasalle, Livingston, Logan, McDonough, McHenry, McLean, Macoupin, Madison, Menard, Peoria, Perry, Piatt, Putnam, Rock Island, St. Clair, Sangamon, Schuyler, Stark, Tazewell, Vermilion, Wayne, Will, and Winn). Data for Pennsylvania are for violent deaths that occurred in 40 counties (Adams, Allegheny, Armstrong, Berks, Blair, Bradford, Bucks, Cameron, Cambria, Carbon, Centre, Chester, Clarion, Clearfield, Clinton, Crawford, Dauphin, Delaware, Erie, Fayette, Forest, Greene, Indiana, Jefferson, Lackawanna, Lancaster, Lehigh, Luzerne, Monroe, Montgomery, Northampton, Philadelphia, Schuylkill, Somerset, Sullivan, Susquehanna, Union, Westmoreland, Wyoming, and York). Data for California are for violent deaths that occurred in 30 counties (Amador, Butte, Colusa, Fresno, Glenn, Humboldt, Imperial, Kern, Kings, Lassen, Lake, Los Angeles, Marin, Modoc, Mono, Orange, Placer, Sacramento, San Benito, San Francisco, San Mateo, Santa Cruz, Shasta, Siskiyou, Solano, Sonoma, Tehama, Trinity, Ventura, and Yolo). Denominators for the rates for these three states (Illinois, Pennsylvania, and California) represent only the populations of the counties from which the data were collected.

** Rates are not reported when number of decedents is <20 or when characteristic response is “other” or “unknown.”

^††^ Includes persons of any race.

^§§^ Other location includes (in descending order): industrial or construction area; hospital or medical facility; bar or nightclub; preschool, school, college, or school bus; synagogue, church, or temple; supervised residential facility; farm; railroad tracks; bridge; abandoned house, building, or warehouse; and other unspecified location.

#### Method and Location of Injury

A firearm was used in the majority (88.0%) of legal intervention deaths (Table 6). Legal intervention deaths occurred most frequently in a house or apartment (35.5%), followed by a street or highway (25.0%) or a motor vehicle (10.6%).

#### Precipitating Circumstances

Precipitating circumstances were identified in 94.1% of legal intervention deaths (Table 7). Among the legal intervention deaths that were precipitated by another crime (78.1%), a crime was reportedly in progress at the time of the incident in almost three fourths (70.0%) of the events (Table 7). When a specific type of crime was known to have precipitated a legal intervention death, the type of crime was most frequently assault or homicide (63.2%), followed by other crime (34.4%), robbery (10.9%), motor vehicle theft (7.6%), burglary (4.5%), rape or sexual assault (2.5%), and drug trade (1.8%) (Supplementary Table S13, https://stacks.cdc.gov/view/cdc/116311). The decedent reportedly used a weapon in 70.7% of legal intervention death cases (Table 7). In 29.6% of legal intervention deaths with known circumstances, a substance use problem (other than alcohol) was reported as a contributing factor, and 19.0% of decedents reportedly had a current diagnosed mental health problem. An argument or conflict and physical fight precipitated 17.3% and 11.2% of legal intervention deaths, respectively. A recent or impending crisis during the previous or upcoming 2 weeks was reported in 7.4% of legal intervention deaths. Among legal intervention deaths with known circumstances, intimate partner violence (9.9%), being a perpetrator of interpersonal violence during the past month (10.2%), family relationship problems (7.1%), and drug involvement (4.7%) were other notable precipitating circumstances.

**TABLE 7:** Number* and percentage^†^ of legal intervention^§^ deaths, by decedent’s sex and precipitating circumstances — National Violent Death Reporting System, 42 states and the District of Columbia,^¶^ 2019

Precipitating circumstance	Male	Female	Total
	No. (%)	No. (%)	No. (%)
**Mental health problems or substance use**			
Substance use problem (excludes alcohol)	188 (29.6)	7 (30.4)	**195 (29.6)**
Current diagnosed mental health problem	117 (18.4)	8 (34.8)	**125 (19.0)**
History of ever being treated for a mental health problem	85 (13.4)	4 (17.4)	**89 (13.5)**
Alcohol problem	70 (11.0)	2 (8.7)	**72 (10.9)**
Current mental health treatment	44 (6.9)	4 (17.4)	**48 (7.3)**
Current depressed mood	22 (3.5)	3 (13.0)	**25 (3.8)**
Other addiction (e.g., gambling or sex)	3 (<1.0)	3 (13.0)	**6 (<1.0)**
**Interpersonal factors**			
Perpetrator of interpersonal violence during past month	66 (10.4)	1 (4.3)	**67 (10.2)**
Intimate partner violence related	64 (10.1)	1 (4.3)	**65 (9.9)**
Family relationship problem	46 (7.2)	1 (4.3)	**47 (7.1)**
Other relationship problem (nonintimate)	22 (3.5)	0 (0.0)	**22 (3.3)**
Victim of interpersonal violence during past month	3 (<1.0)	0 (0.0)	**3 (<1.0)**
Jealousy (lovers’ triangle)	3 (<1.0)	0 (0.0)	**3 (<1.0)**
**Life stressors**			
Argument or conflict	111 (17.5)	3 (13.0)	**114 (17.3)**
Physical fight (two persons, not a brawl)	72 (11.3)	2 (8.7)	**74 (11.2)**
Crisis during previous or upcoming 2 weeks	48 (7.6)	1 (4.3)	**49 (7.4)**
History of child abuse or neglect	4 (<1.0)	0 (0.0)	**4 (<1.0)**
**Crime and criminal activity**			
Precipitated by another crime	497 (78.3)	17 (73.9)	**514 (78.1)**
Crime in progress**	351 (70.6)	9 (52.9)	**360 (70.0)**
Drug involvement	30 (4.7)	1 (4.3)	**31 (4.7)**
Gang related	4 (<1.0)	0 (0.0)	**4 (<1.0)**
**Homicide circumstance**			
Victim used a weapon	450 (70.9)	15 (65.2)	**465 (70.7)**
Stalking	4 (<1.0)	0 (0.0)	**4 (<1.0)**
Victim was a bystander	3 (<1.0)	0 (0.0)	**3 (<1.0)**
Brawl	2 (<1.0)	0 (0.0)	**2 (<1.0)**
Random violence	2 (<1.0)	0 (0.0)	**2 (<1.0)**
Victim was an intervener assisting a crime victim	1 (<1.0)	0 (0.0)	**1 (<1.0)**
Mentally ill suspect	1 (<1.0)	0 (0.0)	**1 (<1.0)**
Hate crime	1 (<1.0)	0 (0.0)	**1 (<1.0)**
Caretaker abuse or neglect led to death	1 (<1.0)	0 (0.0)	**1 (<1.0)**
Prostitution	1 (<1.0)	0 (0.0)	**1 (<1.0)**
**Total^††^**	**635 (94.6)**	**23 (82.1)**	**658 (94.1)**

* Includes deaths with one or more precipitating circumstances. Total numbers do not equal the sums of the columns because more than one circumstance could have been present per decedent.

^†^ Denominator includes those deaths with one or more precipitating circumstances. The sums of percentages in columns exceed 100% because more than one circumstance could have been present per decedent.

^§^ The term “legal intervention” does not denote the lawfulness or legality of the circumstances surrounding the death.

^¶^ Alabama, Alaska, Arizona, California, Colorado, Connecticut, Delaware, District of Columbia, Georgia, Hawaii, Illinois, Indiana, Iowa, Kansas, Kentucky, Louisiana, Maine, Maryland, Massachusetts, Michigan, Minnesota, Missouri, Montana, Nebraska, Nevada, New Hampshire, New Jersey, New Mexico, North Carolina, North Dakota, Ohio, Oklahoma, Oregon, Pennsylvania, Rhode Island, South Carolina, Utah, Vermont, Virginia, Washington, West Virginia, Wisconsin, and Wyoming. Illinois and Pennsylvania collected data on ≥80% of violent deaths in their state, in accordance with requirements under which these states were funded. Data for Illinois are for violent deaths that occurred in 47 counties (Adams, Alexander, Bond, Boone, Brown, Bureau, Champaign, Clay, Cook, DeKalb, Douglas, DuPage, Effingham, Fayette, Fulton, Grundy, Henry, Iroquois, Jackson, Jefferson, Kane, Kankakee, Kendall, Lake, Lasalle, Livingston, Logan, McDonough, McHenry, McLean, Macoupin, Madison, Menard, Peoria, Perry, Piatt, Putnam, Rock Island, St. Clair, Sangamon, Schuyler, Stark, Tazewell, Vermilion, Wayne, Will, and Winn). Data for Pennsylvania are for violent deaths that occurred in 40 counties (Adams, Allegheny, Armstrong, Berks, Blair, Bradford, Bucks, Cameron, Cambria, Carbon, Centre, Chester, Clarion, Clearfield, Clinton, Crawford, Dauphin, Delaware, Erie, Fayette, Forest, Greene, Indiana, Jefferson, Lackawanna, Lancaster, Lehigh, Luzerne, Monroe, Montgomery, Northampton, Philadelphia, Schuylkill, Somerset, Sullivan, Susquehanna, Union, Westmoreland, Wyoming, and York). Data for California are for violent deaths that occurred in 30 counties (Amador, Butte, Colusa, Fresno, Glenn, Humboldt, Imperial, Kern, Kings, Lassen, Lake, Los Angeles, Marin, Modoc, Mono, Orange, Placer, Sacramento, San Benito, San Francisco, San Mateo, Santa Cruz, Shasta, Siskiyou, Solano, Sonoma, Tehama, Trinity, Ventura, and Yolo).

** Denominator includes those decedents involved in an incident that was precipitated by another crime.

^††^ Circumstances were unknown for 41 decedents (36 males and five females); total number of legal intervention deaths = 699 (671 males and 28 females).

### Unintentional Firearm Deaths

#### Sex, Age Group, and Race and Ethnicity

In 2019, a total of 42 NVDRS states (39 states collecting statewide data, 30 California counties, 47 Illinois counties, and 40 Pennsylvania counties) and the District of Columbia collected data on 333 incidents involving 335 unintentional firearm deaths (Supplementary Table S1, https://stacks.cdc.gov/view/cdc/116311). Half (n = 167; 49.9%; data not shown) of these deaths were self-inflicted, and 109 deaths (32.5%; data not shown) were known to be inflicted by another person; for the remaining 59 deaths (17.6%; data not shown), whether the injury was inflicted by the decedent or by another person was unknown. Males accounted for 86.6% of decedents (Table 8). Persons aged ≤24 years accounted for approximately half (50.7%) of all unintentional firearm deaths. The majority of decedents were White persons (55.5%), followed by Black persons (29.6%).

**TABLE 8:** Number and percentage* of unintentional firearm deaths, by selected demographic characteristics of decedent, location of injury, and type of firearm — National Violent Death Reporting System, 42 states and the District of Columbia,^†^ 2019

Characteristic	No. (%)
**Sex**	
Male	290 (86.6)
Female	45 (13.4)
**Race and ethnicity**	
White, non-Hispanic	186 (55.5)
Black, non-Hispanic	99 (29.6)
American Indian or Alaska Native, non-Hispanic	4 (1.2)
Asian or Pacific Islander	5 (1.5)
Hispanic^§^	41 (12.2)
**Age group (yrs)**	
<1	2 (<1.0)
1–4	17 (5.1)
5–9	14 (4.2)
10–14	23 (6.9)
15–19	66 (19.7)
20–24	48 (14.3)
25–29	23 (6.9)
30–34	22 (6.6)
35–44	35 (10.4)
45–54	31 (9.3)
55–64	29 (8.7)
65–74	12 (3.6)
75–84	10 (3.0)
≥85	3 (<1.0)
**Location of injury**	
House or apartment	233 (69.6)
Motor vehicle	26 (7.8)
Natural area	25 (7.5)
Street or highway	8 (2.4)
Hotel or motel	6 (1.8)
Parking lot, public garage, or public transport	5 (1.5)
Other location^¶^	13 (3.9)
Unknown	19 (5.7)
**Firearm type**	
Handgun	213 (63.6)
Rifle	35 (10.4)
Shotgun	30 (9.0)
Other firearm	1 (<1.0)
Unknown	56 (16.7)
**Total**	**335 (100)**

* Percentages might not total 100% due to rounding.

^†^ Alabama, Alaska, Arizona, California, Colorado, Connecticut, Delaware, District of Columbia, Georgia, Hawaii, Illinois, Indiana, Iowa, Kansas, Kentucky, Louisiana, Maine, Maryland, Massachusetts, Michigan, Minnesota, Missouri, Montana, Nebraska, Nevada, New Hampshire, New Jersey, New Mexico, North Carolina, North Dakota, Ohio, Oklahoma, Oregon, Pennsylvania, Rhode Island, South Carolina, Utah, Vermont, Virginia, Washington, West Virginia, Wisconsin, and Wyoming. Illinois and Pennsylvania collected data on ≥80% of violent deaths in their state, in accordance with requirements under which these states were funded. Data for Illinois are for violent deaths that occurred in 47 counties (Adams, Alexander, Bond, Boone, Brown, Bureau, Champaign, Clay, Cook, DeKalb, Douglas, DuPage, Effingham, Fayette, Fulton, Grundy, Henry, Iroquois, Jackson, Jefferson, Kane, Kankakee, Kendall, Lake, Lasalle, Livingston, Logan, McDonough, McHenry, McLean, Macoupin, Madison, Menard, Peoria, Perry, Piatt, Putnam, Rock Island, St. Clair, Sangamon, Schuyler, Stark, Tazewell, Vermilion, Wayne, Will, and Winn). Data for Pennsylvania are for violent deaths that occurred in 40 counties (Adams, Allegheny, Armstrong, Berks, Blair, Bradford, Bucks, Cameron, Cambria, Carbon, Centre, Chester, Clarion, Clearfield, Clinton, Crawford, Dauphin, Delaware, Erie, Fayette, Forest, Greene, Indiana, Jefferson, Lackawanna, Lancaster, Lehigh, Luzerne, Monroe, Montgomery, Northampton, Philadelphia, Schuylkill, Somerset, Sullivan, Susquehanna, Union, Westmoreland, Wyoming, and York). Data for California are for violent deaths that occurred in 30 counties (Amador, Butte, Colusa, Fresno, Glenn, Humboldt, Imperial, Kern, Kings, Lassen, Lake, Los Angeles, Marin, Modoc, Mono, Orange, Placer, Sacramento, San Benito, San Francisco, San Mateo, Santa Cruz, Shasta, Siskiyou, Solano, Sonoma, Tehama, Trinity, Ventura, and Yolo).

^§^ Includes persons of any race.

^¶^ Other location includes (in descending order): commercial or retail area; industrial or construction area; farm; park, playground, sports, or athletic area; preschool, school, college, or school bus; and other unspecified location.

#### Location of Injury and Firearm Type

Among unintentional firearm deaths, 69.6% occurred in a house or apartment, followed by a motor vehicle (7.8%) or natural area (7.5%) (Table 8). The majority of unintentional firearm deaths involved a handgun (63.6%), followed by a rifle (10.4%) or a shotgun (9.0%). In 16.7% of unintentional firearm deaths, the firearm type was unknown.

#### Context and Circumstances of Injury

The context and circumstances of injury were identified in 88.4% of unintentional firearm deaths (Table 9). Among those with context and circumstance information, the most common context of injury was playing with a gun (39.2%), followed by showing the gun to others (11.1%), cleaning the gun (8.4%), and hunting (7.1%). Almost one fourth (22.3%) of unintentional firearm deaths were caused by a person unintentionally pulling the trigger, 13.9% mistakenly thinking the gun was unloaded, and 9.5% mistakenly thinking the magazine was disengaged.

**TABLE 9:** Number and percentage* of unintentional firearm deaths, by context and circumstances of injury — National Violent Death Reporting System, 42 states and the District of Columbia,^†^ 2019

Characteristic	No. (%)
**Context of injury**	
Playing with gun	116 (39.2)
Showing gun to others	33 (11.1)
Cleaning gun	25 (8.4)
Hunting	21 (7.1)
Loading or unloading gun	18 (6.1)
Target shooting	5 (1.7)
Other context of injury	80 (27.0)
**Circumstances of injury**	
Unintentionally pulled trigger	66 (22.3)
Thought gun was unloaded	41 (13.9)
Thought unloaded, magazine disengaged	28 (9.5)
Gun was mistaken for a toy	11 (3.7)
Gun was dropped	9 (3.0)
Gun fired due to defect or malfunction	6 (2.0)
Gun fired while holstering	6 (2.0)
Thought gun safety was engaged	4 (1.4)
Bullet ricocheted	1 (<1.0)
Gun fired while handling safety lock	1 (<1.0)
Other mechanism of injury	73 (24.7)
**Total^§^**	**296 (88.4)**

* Percentages might exceed 100% because one or more circumstances could have been known per death. Number and percentage are reported when the number of deaths is <5 because no particular circumstance identifies a single death. Denominator includes those deaths with one or more precipitating circumstances.

^†^ Alabama, Alaska, Arizona, California, Colorado, Connecticut, Delaware, District of Columbia, Georgia, Hawaii, Illinois, Indiana, Iowa, Kansas, Kentucky, Louisiana, Maine, Maryland, Massachusetts, Michigan, Minnesota, Missouri, Montana, Nebraska, Nevada, New Hampshire, New Jersey, New Mexico, North Carolina, North Dakota, Ohio, Oklahoma, Oregon, Pennsylvania, Rhode Island, South Carolina, Utah, Vermont, Virginia, Washington, West Virginia, Wisconsin, and Wyoming. Illinois and Pennsylvania collected data on ≥80% of violent deaths in their state, in accordance with requirements under which these states were funded. Data for Illinois are for violent deaths that occurred in 47 counties (Adams, Alexander, Bond, Boone, Brown, Bureau, Champaign, Clay, Cook, DeKalb, Douglas, DuPage, Effingham, Fayette, Fulton, Grundy, Henry, Iroquois, Jackson, Jefferson, Kane, Kankakee, Kendall, Lake, Lasalle, Livingston, Logan, McDonough, McHenry, McLean, Macoupin, Madison, Menard, Peoria, Perry, Piatt, Putnam, Rock Island, St. Clair, Sangamon, Schuyler, Stark, Tazewell, Vermilion, Wayne, Will, and Winn). Data for Pennsylvania are for violent deaths that occurred in 40 counties (Adams, Allegheny, Armstrong, Berks, Blair, Bradford, Bucks, Cameron, Cambria, Carbon, Centre, Chester, Clarion, Clearfield, Clinton, Crawford, Dauphin, Delaware, Erie, Fayette, Forest, Greene, Indiana, Jefferson, Lackawanna, Lancaster, Lehigh, Luzerne, Monroe, Montgomery, Northampton, Philadelphia, Schuylkill, Somerset, Sullivan, Susquehanna, Union, Westmoreland, Wyoming, and York). Data for California are for violent deaths that occurred in 30 counties (Amador, Butte, Colusa, Fresno, Glenn, Humboldt, Imperial, Kern, Kings, Lassen, Lake, Los Angeles, Marin, Modoc, Mono, Orange, Placer, Sacramento, San Benito, San Francisco, San Mateo, Santa Cruz, Shasta, Siskiyou, Solano, Sonoma, Tehama, Trinity, Ventura, and Yolo).

^§^ Circumstances were unknown for 39 decedents; the total number of unintentional firearm decedents = 335.

### Deaths of Undetermined Intent

#### Sex, Age Group, and Race and Ethnicity

In 2019, a total of 42 NVDRS states (39 states collecting statewide data, 30 California counties, 47 Illinois counties, and 40 Pennsylvania counties) and the District of Columbia collected data on 4,452 incidents involving 4,504 deaths of undetermined intent (Supplementary Table S1, https://stacks.cdc.gov/view/cdc/116311). The overall rate of deaths of undetermined intent was 2.0 per 100,000 population (Supplementary Table S4, https://stacks.cdc.gov/view/cdc/116311). The rate of deaths of undetermined intent was higher among males (2.7 per 100,000 population) than among females (1.4 per 100,000 population). Over half (59.8%) of deaths of undetermined intent were among adults aged 35–64 years. The rate of deaths of undetermined intent was highest among adults aged 30–34 years and 35–44 years (both at 3.3 per 100,000 population), followed by adults aged 45–54 years (3.2 per 100,000 population) and 55–64 years (3.0 per 100,000 population), infants (i.e., children aged <1 year; 2.8 per 100,000 population), and adults aged 25–29 years (2.5 per 100,000 population). Although White persons accounted for the majority (65.3%, 2.0 per 100,000 population) of deaths of undetermined intent, Black persons had the highest rate (3.9 per 100,000 population). Among males, Black males (5.8 per 100,000 population) and AI/AN males (4.4 per 100,000 population) had the highest rate of deaths of undetermined intent. Among females, Black and AI/AN females also had the highest rates of deaths of undetermined intent (both 2.2 per 100,000 population), followed by White females (1.5 per 100,000 population).

#### Method and Location of Injury

Poisoning was the most common method of injury in deaths of undetermined intent (71.7%), followed by drowning (4.2%); firearm (4.1%); fall and blunt instrument (2.6% each); motor vehicle (2.2%); fire or burns (1.9%); hanging, strangulation, or suffocation (1.7%); and other (1.2%). Sharp instruments, personal weapons, intentional neglect, and shaking were each used as method of injury in <1.0% of undetermined intent deaths (Supplementary Table S4, https://stacks.cdc.gov/view/cdc/116311). Weapon type was unknown for 6.6% of undetermined intent deaths. The majority of deaths of undetermined intent occurred in a house or apartment (67.0%), followed by a natural area (5.2%), another location (4.5%), or a street or highway (4.2%).

#### Toxicology Results of Decedent

Toxicology tests for BAC were conducted for 76.6% of decedents in deaths of undetermined intent (Supplementary Table S5, https://stacks.cdc.gov/view/cdc/116311). Among those with positive results for alcohol (33.0%), 49.3% had a BAC ≥0.08 g/dL. Tests for the following substances were conducted for the percentage of decedents indicated in parentheses: amphetamines (37.7%), antidepressants (38.3%), benzodiazepines (41.2%), cannabis (more commonly referred to as marijuana; 32.9%), cocaine (50.1%), and opioids (74.0%). Among decedents tested for amphetamines, 28.6% had positive test results. Among those tested for antidepressants, 52.4% had positive results at the time of death, 41.5% of those tested for benzodiazepines had positive results, 27.5% of those tested for cannabis had positive results, and 43.2% of those tested for cocaine had positive results. Results for opioids (including illicit or prescription) were positive in 77.1% of decedents tested. Carbon monoxide was tested for a substantially smaller proportion of decedents (7.0%) but was identified in 37.8% of those decedents.

#### Precipitating Circumstances

Circumstances were identified in 82.6% of deaths of undetermined intent (Supplementary Table S6, https://stacks.cdc.gov/view/cdc/116311). Among deaths of undetermined intent with known circumstances, 35.5% of decedents had a current diagnosed mental health problem at time of death; depression or dysthymia (58.9%), anxiety disorder (28.3%), and bipolar disorder (20.1%) were the most common diagnoses among these decedents. Among all deaths of undetermined intent, 24.2% were receiving mental health treatment at the time of death, and 8.0% of decedents had a current depressed mood. Substance use problems (other than alcohol; 71.1%), history of ever being treated for a mental health problem (32.2%), and alcohol problems (25.8%) were the most commonly reported circumstances. Physical health problems (10.8%) and a recent or impending crisis during the preceding or upcoming 2 weeks (8.2%) were other life stressors identified in deaths of undetermined intent. Among decedents, 10.8% had a history of suicidal thoughts or plans, 7.5% had a history of attempting suicide, and 4.9% had disclosed intent to die by suicide.

### Violent Deaths in Puerto Rico

For 2019, Puerto Rico collected data on 831 incidents involving 897 deaths (data not shown). Homicide (n = 632) accounted for the largest proportion (70.5%) and highest rate (19.8 per 100,000 population) of violent deaths, followed by suicide (n = 229; 25.5%; 7.8 per 100,000 population aged ≥10 years) (Supplementary Table S15, https://stacks.cdc.gov/view/cdc/116311).

#### Homicides

##### Sex, Age Group, and Race and Ethnicity

In 2019, a total of 591 homicides among males and 41 homicides among females were reported in Puerto Rico (Supplementary Table S15, https://stacks.cdc.gov/view/cdc/116311). The overall homicide rate for males (39.0 per 100,000 population) was 16.3 times the rate for females (2.4 per 100,000 population). Among males, the homicide rate was 100.3 per 100,000 population among adults aged 18–29 years and 73.9 per 100,000 population among those aged 30–44 years. Most (96.0%) homicide victims were Hispanic.

##### Method, Location of Injury, and Victim-Suspect Relationship

A firearm was used in the majority (91.0%) of homicides (Supplementary Table S15, https://stacks.cdc.gov/view/cdc/116311). A firearm was the most common method used in homicides of both males (92.6%) and females (68.3%); however, the firearm homicide rate for males (36.1 per 100,000 population) was 21.2 times the rate for females (1.7 per 100,000 population). Among males, a street or highway was the most common location (48.2%) of homicides, whereas a house or apartment was the most common location (43.9%) of homicides for females.

The victim-suspect relationship was known for 38.4% of homicides (Supplementary Table S15, https://stacks.cdc.gov/view/cdc/116311). When the relationship was known, the suspect for male victims was most often a person known to the victim, but the exact nature of the relationship was unclear (53.0%), whereas the suspect for almost two thirds (58.3%) of female victims was a current or former intimate partner.

##### Toxicology Results of Decedent

Tests for BAC were conducted for 99.4% of homicide decedents (Supplementary Table S16, https://stacks.cdc.gov/view/cdc/116311). Among those with positive results for alcohol (32.6%), 45.9% had a BAC ≥0.08 g/dL. Tests for cannabis (more commonly referred to as marijuana), cocaine, and opioids were conducted for 61.6%, 99.5%, and 99.4% of decedents, respectively. Results for cannabis, cocaine, and opioids were positive in 22.1%, 12.6%, and 3.3% of decedents tested, respectively.

##### Precipitating Circumstances

Precipitating circumstances were identified in 92.9% of homicides (Supplementary Table S17, https://stacks.cdc.gov/view/cdc/116311). Among males, 53.9% of homicides involved illicit drugs, 40.4% were gang related, and almost one fourth (22.5%) involved drive-by shootings. Intimate partner violence was identified as a contributing factor in 7.2% of homicides overall. Almost half (42.5%) of homicides among females were precipitated by intimate partner violence, whereas 4.6% of homicides among males were precipitated by intimate partner violence.

#### Suicides

##### Sex, Age Group, and Race and Ethnicity

In 2019, a total of 229 suicides among persons aged ≥10 years (185 suicides among males and 44 suicides among females) were reported in Puerto Rico (Supplementary Table S18, https://stacks.cdc.gov/view/cdc/116311). The suicide rate for males was 4.7 times the rate for females (13.5 versus 2.9 per 100,000 population aged ≥10 years). The suicide rates were highest among men aged 30–44 years (13.9 per 100,000), 45–64 years (19.2 per 100,000), and ≥65 years (17.3 per 100,000 population). The majority (94.3%) of suicide decedents overall were Hispanic.

##### Method and Location of Injury

Hanging, strangulation, or suffocation was the most commonly used method for suicide among both males (69.7%) and females (61.4%) (Supplementary Table S18, https://stacks.cdc.gov/view/cdc/116311). A firearm was used in 17.3% of suicides among males. The most common location where a suicide took place was a house or apartment both for males (82.7%) and females (88.6%).

##### Toxicology Results of Decedent

Tests for BAC were conducted for 98.7% of suicide decedents (Supplementary Table S19, https://stacks.cdc.gov/view/cdc/116311). Among those with positive results for alcohol (31.0%), 44.3% had a BAC ≥0.08 g/dL. Other than alcohol, suicide decedents were most often tested for cannabis (more commonly referred to as marijuana; 50.7%), cocaine (99.1%), and opioids (99.1%). Results for cocaine were positive in 10.1% of decedents tested.

##### Precipitating Circumstances

Circumstances were identified in 90.8% of suicides (Supplementary Table S20, https://stacks.cdc.gov/view/cdc/116311). Overall, a mental health problem was the most common circumstance among suicide decedents, with 56.3% having a current diagnosed mental health problem and 39.4% experiencing a depressed mood at the time of death.

Among males, 40.4% of suicide decedents had a current depressed mood, and 50.6% had a current diagnosed mental health problem. Depression or dysthymia was most often the mental health diagnosis experienced by male suicide decedents with a diagnosed mental health problem (69.0%), followed by schizophrenia (23.8%). One third (34.9%) of male suicide decedents had a history of ever being treated for a mental health problem. Almost one third (28.9%) of male suicide decedents had a history of expressing suicidal thoughts and plans, and 37.3% had a history of attempting suicide. Other precipitating circumstances for male suicide decedents included intimate partner problems (14.5%), physical health problem (13.9%), and financial problem (6.6%).

Among female suicide decedents, 35.7% had a current depressed mood, and 78.6% had a current diagnosed mental health problem. Depression or dysthymia was most often the mental health diagnosis experienced by female suicide decedents who had a diagnosed mental health problem (78.8%). Over half (57.1%) of female decedents had a history of ever being treated for a mental health problem, and almost one third (31.0%) had a history of attempting suicide.

## Discussion

Violent deaths affect all subgroups, regardless of sex, age, or race and ethnicity. NVDRS provides information on specific manners of violent death and can be used to describe characteristics of and inequities experienced by populations particularly affected by fatal violence. NVDRS data can also identify common risk factors for multiple forms of violence. These details increase the knowledge base about the circumstances associated with violence and can assist public health authorities and their partners in developing and guiding effective, data-driven approaches to violence prevention.

The occurrence of violent death varies greatly across states, the District of Columbia, and Puerto Rico (*1*). This report summarizes data on violent deaths that occurred in 2019 in 42 NVDRS states, the District of Columbia, and Puerto Rico, and describes selected characteristics. The 42 states and the District of Columbia represented 67.7% of the U.S. population (*7*) and accounted for 70.3% of violent deaths in the United States in 2019 (*1*). In 2019, NVDRS expanded data collection to include all 50 states, the District of Columbia, and Puerto Rico, providing more comprehensive, accessible, and actionable violent death information that can be used to guide the development of evidence-based violence prevention efforts at local, regional, state, and national levels. Expanding NVDRS to a nationwide system also contributes to the national prevention initiative *Healthy People 2020* objectives to increase the number of states that link data on violent deaths from death certificates, coroner or medical examiner records, and law enforcement reports at state and local levels and the *Healthy People 2030* objectives to reduce the number of suicides, homicides, and firearm-related deaths (*12*,*13*).

Violence is preventable and reducing violent deaths in communities is possible with evidence-based approaches (*14*). CDC developed technical packages to assist communities in identifying violence prevention approaches that are based on the best available evidence. The five technical packages describe strategies, approaches, and specific programs, practices, and policies with evidence to reduce the risk for suicide, youth violence, child abuse and neglect, intimate partner violence, and sexual violence. Each technical package considers the multifaceted and interactive effects of different levels of the social ecology, including individual, relationship, family, school, and community factors that influence violence-related outcomes. NVDRS gathers ongoing, systematic, and consistent data on violent deaths that can be used by violence prevention experts within their communities to guide planning and implementation and track outcomes of violence prevention strategies and approaches, such as those described in CDC’s technical packages for violence prevention.

### Suicides

#### Suicide Circumstances

Suicide rates were highest among males and adults aged 45–64 years. Approximately one third of suicide decedents had a history of suicidal thoughts or plans, and almost one fourth had disclosed their suicidal intent. Multiple factors contribute to the risk for suicide (*15*), and the findings in this report indicate that intimate partner problems, recent or impending crises, arguments or conflicts, job problems, and physical and mental health problems were common precipitating circumstances. Mental health problems were the most commonly identified circumstance, yet approximately half of suicide decedents did not have a known diagnosed mental health condition at the time of their death. Past suicidal behavior and mental health problems are well documented as important risk factors to target in suicide prevention (*16*,*17*). Only one fourth of suicide decedents were known to be receiving treatment at the time of death, pointing to a gap between those receiving treatment and those who would likely benefit from it. Mental health problems and substance use often co-occur among suicide victims (*18*).

In this analysis, a high prevalence of alcohol use, especially BAC ≥0.08 g/dL, was observed among suicide decedents who were tested for substances. Alcohol use is a known predictor of suicidal behavior (*19*), victimization (*20*), and interpersonal violence perpetration (*21*,*22*). Intoxication can cause disinhibition, enhanced feelings of hopelessness and depression, and impaired judgment, which can lead to impulsive behaviors (*17*). Positive toxicology results for opioids (illicit or prescription) were reported in almost one fourth of suicide decedents tested for these substances. Opioid overdose has been recognized as an epidemic (*23*). As a result, CDC has implemented comprehensive surveillance and prevention activities through the Overdose Data to Action cooperative agreement to support state and local health departments in collecting and reporting more timely and complete data on overdose morbidity and mortality and using the data to guide prevention and response efforts (*24*–*26*). In addition, previous research suggests that chronic pain might be a contributor to suicide (*27*). The *CDC Guideline for Prescribing Opioids for Chronic Pain* aims to ensure patients receive appropriate care for pain by providing recommendations for primary care physicians who are prescribing opioids for chronic pain outside of active cancer treatment, palliative care, and end-of-life care to improve communication between clinicians and patients about the risks and benefits of opioid therapy for chronic pain, supporting safer prescribing practices, and reducing risks associated with long-term opioid therapy, including opioid use disorder, prescription opioid misuse, overdose, and death (*28*). Other important activities to address the opioid overdose epidemic include expanding naloxone availability and access to treatment with medications for opioid use disorder, addressing prescription opioid misuse, enhancing public health and public safety partnerships, and maximizing the ability of health systems to link persons who use drugs to treatment and harm-reduction services (*24*–*27*,*29*). Another factor that might contribute to the risk for suicide is access to lethal means among persons at risk for suicide (*16*). The most common method used in suicides was a firearm. Lethal means, such as firearms, provide limited opportunity for intervention and have high case-fatality rates (*16*). Creating protective environments by reducing access to lethal means among persons at risk can be an effective strategy to prevent suicide (*16*).

#### Racial and Ethnic Inequities in Suicide Rates

Demographic variations persist in the manner of death from violence-related injuries. Suicides comprised the majority of violent deaths collected in NVDRS and occurred at higher rates among AI/AN and White persons compared with other racial and ethnic groups. The findings regarding suicide rates experienced by AI/AN persons, in particular, warrant attention to the contextual factors that might contribute to higher rates of suicide, such as barriers to accessing mental health care, exposure to the suicide of a friend or family member as a contributing factor to a person’s own death by suicide, and alcohol and substance use (*30*). Previous studies have described how experiences with historical trauma among AI/AN persons related to the intergenerational, collective, and cumulative impact of colonialism and ongoing inequities including discrimination, disparaging stereotypes, and microaggressions can contribute to risk for suicide (*31*,*32*). Challenges related to suicide, alcohol, and substance use are not inherent to AI/AN culture but can be interpreted within the context of historical racism and ongoing inequities. Furthermore, acknowledging the heterogeneity among persons and groups who identify as AI/AN is important (*30*,*31*).

#### Suicide Prevention Strategies

Participating NVDRS programs have partnered with collaborators to use VDRS data to examine violent deaths and guide prevention efforts, with several states using their VDRS data to guide suicide prevention efforts within their respective states. For example, VDRS programs in New Hampshire, Indiana, and Colorado have used their local data to guide suicide prevention efforts and generate reports highlighting where additional focus for suicide prevention is needed. New Hampshire VDRS data have been used to monitor suicide rates and guide statewide collaborative prevention efforts (*33*). For data years 2014–2018, the crude suicide rate in New Hampshire was 18.8 per 100,000 persons of all ages, compared with 14.1 per 100,000 for persons of all ages in the United States. The National Alliance on Mental Illness New Hampshire, State Suicide Prevention Council, and Youth Suicide Prevention Assembly used New Hampshire VDRS data as the primary data source for its 2019 Suicide Prevention Annual Report, which highlights areas of interest or concern in the state and success stories from statewide suicide prevention initiatives (*33*). Data from the Indiana VDRS indicated that in 2017, Black persons died by suicide at a higher rate in Indiana than in the United States overall (8.4 versus 6.6 per 100,000) (*34*). In addition, among Black persons who died by suicide in Indiana, 14.6% were aged ≤18 years, compared with 3.6% of White decedents (*34*). These findings highlight the connection between suicide and mental health–related problems disproportionately experienced among Black persons in Indiana, underscoring a need for improved suicide awareness and culturally competent mental health care to guide suicide prevention efforts. In Colorado, through a partnership with the Colorado National Collaborative, Colorado VDRS data were used to identify regions with high indicators of suicide-related risk factors or outcomes (*35*). Geospatial and demographic analysis, paired with an environmental scan of existing prevention efforts and resources, allowed Colorado to better understand the contexts in which suicides can be prevented, including risk and protective factors, community needs, capacity for suicide prevention, and available community-based preventive services (*35*). Through this effort, Colorado identified six counties with the highest suicide rates and worked with community coalitions to implement a suicide prevention technical package of evidence-driven strategies (*35*). The Colorado technical package, modeled after CDC’s suicide prevention technical package, includes six components: 1) increase connectedness (social and structural), 2) increase economic stability and support, 3) increase education and awareness, 4) increase access to suicide safer care (for persons at risk), 5) improve lethal means safety, and 6) increase services for postventions (i.e., interventions conducted after a suicide for those affected by the death) (*35*). This collaboration highlights the importance of using the data to identify priority populations and mobilize comprehensive, coordinated efforts by national, state, and local partners in implementing evidence-based programs and interventions to improve the health and well-being of persons in high-rate counties and prevent suicide.

CDC’s suicide prevention technical package describes the following seven strategies for reducing suicide and suicidal behaviors: 1) strengthen economic supports, 2) strengthen access to and delivery of suicide care, 3) create protective environments, 4) promote connectedness, 5) teach coping and problem-solving skills, 6) identify and support persons at risk, and 7) decrease harms and prevent future risk (*14*). These strategies support the goals and objectives of the National Strategy for Suicide Prevention (NSSP), a comprehensive national agenda for suicide prevention (*36*), and the National Action Alliance for Suicide Prevention’s priority to strengthen community-based prevention (*37*). NVDRS is relevant to the NSSP goals of increasing timeliness and usefulness of surveillance systems related to suicide prevention and evaluating outcomes and effectiveness of suicide prevention interventions. The suicide prevention technical package includes examples of specific approaches that communities can implement to use each strategy. The findings in this report underscore the importance of approaches outlined in the suicide technical package, such as social-emotional learning programs, enhanced parenting skills and family relationships, and treatment for persons at risk for suicide and treatment to prevent reattempts.

### Homicides

#### Homicides of Infants and Children

Although homicide rates for children varied significantly by age, in this analysis, infants (i.e., children aged <1 year) had a higher homicide rate than children aged 1–14 years. Some studies have found the highest risk for infant homicide is on the day of birth (*38*,*39*). Risk starts in infancy and continues throughout childhood, highlighting the need to prioritize child abuse and neglect prevention and intervention strategies to reduce risk for morbidity and mortality (*40*). Child abuse and neglect are often associated with immediate physical injuries, emotional and psychological problems, involvement in risky health behaviors later in life, and a wide range of broader physical health challenges and long-term health consequences (*40*).

CDC’s child abuse and neglect prevention technical package identified the following evidence-based strategies and approaches: 1) strengthening economic supports for families, 2) changing social norms to support parents and positive parenting, 3) providing quality care and education early in life, 4) enhancing parenting skills to promote healthy child development, and 5) intervening to decrease harms and prevent future risk (*40*). Deaths among children related to child abuse and neglect are preventable, and the specific approaches described in the technical package can help create safe, stable, and nurturing relationships and environments (*41*) to prevent homicides among infants and children as well as nonfatal child abuse and neglect. Many children do not have these safe and stable relationships and environments, which are essential for promoting children’s health and well-being, and are therefore at risk for adverse childhood experiences (e.g., violence, abuse, or death). CDC’s comprehensive approach to preventing adverse childhood experiences uses multiple approaches, such as promoting public education campaigns on social norms to support parents and positive parenting, ensuring a strong start for children through programs such as early childhood home visitation, quality and affordable child care, and preschool enrichment programs, decreasing risk for child abuse and neglect-related fatalities (*42*).

#### Racial and Ethnic Inequities in Homicide Rates

Racial and ethnic minority groups experience inequitable rates of violent injury and homicide, particularly among youths and young adult males (*43*). Black and AI/AN persons experienced the highest rates of homicide in the U.S. states and the District of Columbia. Similarly, among females, the homicide rate was highest among Black and AI/AN persons. In Puerto Rico, the homicide rate was approximately double the suicide rate, and males in Puerto Rico experienced homicide rates similar to and exceeding the homicide rates experienced by AI/AN and Hispanic males in the U.S. states and the District of Columbia. Racial and ethnic inequities in exposure to violence are pervasive and persistent, and the elimination of these inequities should be prioritized (*43*). Racial and ethnic minority groups are disproportionately exposed to systemic inequities such as residential segregation, concentrated disadvantage, stress from experiencing racism, limited access to the best educational and employment opportunities, and other conditions that increase the risk for experiencing violence (*44*,*45*). For example, homicide rates for males in Puerto Rico have been attributed, in part, to living in communities that have been marginalized and the socioeconomic incentives of being involved in illegal means of income that are associated with high risks for violence (*46*). Racial and ethnic minority youths often live in communities with concentrated poverty, stressed economies, residential instability, neighborhood disorganization, low community cohesion, and informal controls (*44*,*45*,*47*). All of these conditions are associated with violence and violence-related injuries, and addressing the contextual factors at the structural, societal, and community levels that serve as risk factors can have broad and sustained effects in preventing violence (*3*,*44*,*45*,*47*).

#### Homicide Prevention Strategies

NVDRS programs have used their local VDRS data to examine disparities related to homicide in their states. For example, the North Carolina VDRS program examined homicide rates for 2019 and found the homicide rates were highest among AI/AN persons (17.6 per 100,000) and Black persons (17.4 per 100,000) compared with other racial and ethnic groups (*48*). In addition, the homicide rates among AI/AN and Black persons were approximately 2.5 times higher than the statewide homicide rate (*48*). Recognizing this disparity, the North Carolina VDRS program, in collaboration with the University of North Carolina (UNC) Injury Prevention Research Center (IPRC), expanded their data workshop training program to increase awareness and promote use of North Carolina VDRS data at state universities and colleges. After a data workshop conducted with UNC Pembroke, a school with a history of serving the local Lumbee American Indian population, the North Carolina VDRS program and UNC IPRC partnered with UNC Pembroke to train social work students to use VDRS data to address health equity issues in and around their immediate community. Using VDRS as the main data source, the North Carolina VDRS program aims to expand partnerships to include historically Black colleges and universities in the state to empower researchers to use VDRS data to address health equity issues in and around their immediate community.

In conjunction with other data sources, NVDRS data can be used to can help states identify and target salient factors related to violence at the neighborhood and community levels, which can contribute to greater population-level decreases in violence through the reduction and elimination of systemic inequities (*47*). CDC’s youth violence prevention technical package outlines several programs and approaches at the community and societal levels (*14*), such as Baltimore’s Safe Streets (*49*), Crime Prevention Through Environmental Design (*50*), business improvement districts (*51*,*52*), and policies such as the Earned Income Tax Credit (EITC) (*53*,*54*). For example, enhancing household financial security through tax credits such as the EITC can help families increase their income while providing an incentive to have a job or counterbalancing the costs of child-rearing and help create home environments that encourage healthy development (*53*,*54*). Evaluations of these programs and policies have confirmed the value of using these types of approaches to reduce the risk for violence and promote protective community environments (*14*). Evidence also suggests that these approaches and other universal policies that focus on general community improvements can have a substantial impact on decreasing racial and ethnic inequities in violence (*40*).

The disproportionate homicide risk among AI/AN females has garnered national and political attention given the underreporting of missing and murdered indigenous women in the United States (*55*–*57*). For example, in 2016, the National Crime Information Center recorded 5,712 reports of missing AI/AN women and girls, whereas the U.S. Department of Justice had only 116 such cases recorded in the same year (*55*). Two laws, Savanna’s Act and the Not Invisible Act, were recently enacted to provide legal provisions to increase and improve data on the number of missing or murdered AI/AN persons, including AI/AN women and girls (*58*,*59*). Approaches that improve data collection (e.g., improve racial classification of records and record-keeping among law enforcement) and relationships between journalists and AI/AN communities have been noted as meaningful ways to address violence against AI/AN women and girls (*55*).

#### Intimate Partner Violence–Related Homicides

Intimate partner violence–related homicides among males were most often precipitated by an argument or conflict or during the commission of a crime (predominately assault or homicide). In contrast, approximately 45.3% of homicides among females were related to intimate partner violence, and a current or former intimate partner was identified as the suspect for approximately half of female homicide victims with known suspects. These findings were consistent with another NVDRS report that highlighted the differential impact of intimate partner violence–related homicides among young and racial and ethnic minority women (*60*). Estimates from the 2015 National Intimate Partner and Sexual Violence Survey indicate that approximately 80 million persons in the United States have experienced intimate partner violence (e.g., contact sexual violence, physical violence, or stalking by an intimate partner) at some point in their lives, and approximately 12 million within the previous 12 months (*61*). Intimate partner violence–related homicides warrant further research to determine the contextual factors and characteristics of these fatal incidents and how these contextual factors might vary by various demographic characteristics.

NVDRS programs have used their data to examine intimate partner violence–related homicides to support efforts to prevent violence against women. In 2019, Puerto Rico VDRS data revealed that 58.3% of female homicide victims were killed by a current or former spouse or intimate partner, and 42.5% of female homicides were related to intimate partner violence (Supplementary Table S15, https://stacks.cdc.gov/view/cdc/116311). In 2021, the governor of Puerto Rico declared a state of emergency for violence against women and called for the formation of the intergovernmental agency Committee for the Prevention, Support, Rescue, and Education of Gender Violence (PARE in Spanish) to address the ongoing crisis of gender-based violence in Puerto Rico (*62*). Puerto Rico VDRS is supporting the development of an interagency platform by providing data, collected by the Puerto Rico VDRS program, on violence against women.

CDC’s intimate partner violence prevention technical package outlines several strategies for programs and policies to prevent intimate partner–related violence and to decrease harms (*63*). Strategies and approaches to prevent and reduce intimate partner violence might occur across different levels of the social ecology, such as engaging men and boys as allies (*63*,*64*); disrupting developmental pathways toward intimate partner violence; creating protective school, workplace, and neighborhood environments (*63*); teaching youths about safe and healthy relationships (*63*,*65*); empowering bystanders; and strengthening economic supports for families (*63*). Prevention efforts can help change harmful gender norms that condone violence and the societal conditions that serve to maintain those norms (*63*,*66*).

### Legal Intervention Deaths

NVDRS collects more complete information on legal intervention deaths than other existing data sources (*67*). The rate of legal intervention death was highest among AI/AN persons, and the rate among Black males was 3.5 times that of their White male counterparts, a finding consistent with previous studies (*68*,*69*). Racial and ethnic inequities in fatal police shootings have been examined in the literature (*68*,*70*–*72*) and have been found to be associated with factors such as increased police contact due to more traffic stops, higher presence of law enforcement in racial and ethnic minority communities, and race-based bias and perceptions of threat. More analyses are needed to increase knowledge about the magnitude and circumstances of these deaths and for developing appropriate prevention strategies and monitoring their effectiveness. Multiple strategies have been proposed to reform policing as possible ways of decreasing legal intervention deaths (*68*,*70*,*73*–*77*). For example, some studies have suggested increasing training for law enforcement to reduce potential bias in interactions with suspects as well as training in conflict de-escalation and tactical disengagement as approaches to reducing legal intervention deaths (*68*,*70*). Similarly, in a 2020 letter to Congress, the American Medical Association urged for police reform, including steps that might address excessive use of violence against persons in minoritized and marginalized communities, such as training for law enforcement at all levels on implicit or unconscious bias and structural racism, increased use of body-worn cameras by law enforcement officers, and reauthorizing federal programs for juvenile justice and delinquency prevention (*73*). Some governing bodies have passed legislation to hold law enforcement accountable for misconduct by limiting or eliminating qualified immunity (*74*–*77*), a judicial doctrine that protects government officials from lawsuits alleging a violation of a right other than a plaintiff’s clearly established statutory or constitutional right (*78*). The impact of these laws has not yet been studied. In 2016, the U.S. Department of Justice Equal Opportunity Commission engaged in an initiative designed to help U.S. law enforcement agencies recruit, hire, retain, and promote officers who reflect the diversity of the communities they serve, all of which have been found to improve trust and relations between law enforcement and communities (*79*).

A unique strength of the NVDRS surveillance system is the ability to collect data on suspects, including characteristics of law enforcement officers involved in legal intervention deaths (*2*,*80*). Although not examined in the current report, a previous study examining characteristics of officers involved in legal intervention deaths found associations between officer use of lethal force and characteristics such as race, age, sex, education, and previous use of force (*80*). Given previous findings on characteristics of officers involved in legal intervention deaths and the importance of NVDRS for capturing information on legal intervention deaths, researchers have called on NVDRS to increase the completeness of demographic information on officers involved in these deaths (*68*,*80*).

### Unintentional Firearm Deaths

NVDRS also has been recognized as a reliable source of data on unintentional firearm deaths (*81*,*82*) and for its ability to provide details about victims and shooters (*81*,*82*). In this report, approximately half of unintentional firearm deaths were self-inflicted; however, approximately one third were inflicted by another person. Most of these deaths occurred while playing with a gun, accidentally pulling the trigger of a gun, or thinking a gun was unloaded, which are concerning circumstances, particularly among children; these findings highlight the importance of safe storage practices and education about safe handling of firearms (*83*).

## Limitations

The findings in this report are subject to at least seven limitations. First, NVDRS data are available from a limited number of states, the District of Columbia, and Puerto Rico and therefore are not nationally representative. In addition, California, Illinois, and Pennsylvania data were from a subset of counties and are not representative of all violent deaths occurring in these states. However, Illinois and Pennsylvania contributed data that represent a high percentage of the state populations (83% and 90%, respectively), and all of these states include a mix of data from large urban population centers and smaller, more rural counties.

Second, the availability, completeness, and timeliness of data depend on partnerships among VDRS programs and local health departments, vital statistics registrars’ offices, coroners and medical examiners, and law enforcement personnel. Data sharing and communication among partners are particularly challenging when states and U.S. territories have independent county coroner systems (rather than a centralized coroner or medical examiner system), numerous law enforcement jurisdictions, or both. NVDRS incident data might be limited or incomplete for areas in which these data-sharing relations are not fully developed. Partnerships with local vital statistics registrars’ offices usually are more established because they are part of the public health infrastructure. As part of an active surveillance system, VDRS programs work closely with local vital registrars’ offices to identify deaths that meet the NVDRS case definition and to avoid cases being missed or inappropriately included. CDC also monitors case ascertainment and variable completeness through regular technical assistance calls, which include reviews of the internal data quality dashboard in the web-based system that is updated in real time. Overall, core variables that represent demographic characteristics (e.g., age, sex, and race and ethnicity) and manner of death were known for >99% of cases.

Third, toxicology data are not collected consistently across all states, the District of Columbia, and Puerto Rico or for all alcohol and drug categories. In addition, toxicology testing is not conducted for all decedents; thus, percentages of decedents with positive results for specific substances might be affected by testing practices in coroner or medical examiner offices (*84*).

Fourth, abstractors are limited to the data included in the investigative reports they receive. In addition, reports might not fully reflect all information known about an incident, particularly for homicides and legal intervention deaths, when data are less readily available until a full investigation and adjudication are completed.

Fifth, case definitions present challenges when a single death is classified differently in different documents (e.g., unintentional firearm death in a law enforcement report, homicide in a coroner or medical examiner record, and undetermined on the death certificate). NVDRS abstractors reconcile these discrepancies using standard NVDRS case definitions and select a single manner of death on the basis of all source documents (*6*).

Sixth, variations in coding occur depending on the abstractor’s level of experience. For this reason, CDC provides extensive abstractor guidance and training, a coding manual to promote standardized data collection (*6*), and data validation checks. As part of their internal data quality efforts, VDRS programs are required to reabstract at least 5% cases to examine consistency in coding and identify training needs of data abstractors.

Finally, medical and mental health information (e.g., type of condition and whether the decedent was receiving treatment) often are not captured directly from medical records but from coroner or medical examiner records and the decedent’s family members and friends. Therefore, the completeness and accuracy of this information are limited to the knowledge of the informant.

## Future Directions 

As a web-based surveillance system, NVDRS continues to evolve, with recent modifications incorporating several elements. For example, a comprehensive review of toxicology fields in NVDRS was conducted to identify substances that had never been coded. These unused fields were deactivated to allow for more focused data entry (i.e., data reflective of those elements contained in toxicology reports). In addition, substance categories were expanded for more precision. The system already has an auto-fill feature to enhance the abstractor experience and make data entry of substances more efficient. Recently, two new modules were added to NVDRS: 1) a school-associated violent death module that will collect in-depth information to determine circumstances and other contextual factors for such deaths and help guide efforts to prevent fatal school violence and 2) a public safety officer suicide module to capture in-depth information regarding circumstances and other contextual factors specific to first responders who died by suicide. Collecting information in these newly added modules will help develop programs and guide efforts to prevent school-associated violent deaths and suicide among public safety officers. Last, this report summarizes data on violent deaths that occurred in 2019 in 42 NVDRS states, the District of Columbia, and Puerto Rico. The goal is to include data for all 50 states, the District of Columbia, and Puerto Rico in future reports.

## Conclusion

Public health surveillance is the foundation for public health practice (*85*). Monitoring the prevalence of violence-related fatal injuries, defining priorities, and guiding violence prevention activities are essential parts of public health surveillance. In 2018, NVDRS received funding for nationwide expansion. Although not all VDRS programs’ data met the inclusion criteria to be included in this report, as of 2019, all 50 states, the District of Columbia, and Puerto Rico began participating and entering data in NVDRS, an important step toward achieving the ultimate goal of providing nationally representative data. This expansion makes violent death information available for local communities to develop prevention efforts and allow for the system’s capacity to measure the need for and effects of violence prevention policies, programs, and practices at the national level.

## Footnotes

* Frequencies and rates of violent deaths included in this report differ slightly from the frequencies and rates of violent deaths reported by CDC’s WISQARS, which excludes nonresident deaths that occur in participating states and the District of Columbia (i.e., occurrent deaths). NVDRS tracks both resident and occurrent violent deaths in the overall data set, and the numbers in this report reflect both. VDRS programs are expected to collect information on violent deaths among their residents, wherever they occur, and fatal violent injuries occurring within their borders irrespective of the decedent’s residence status. If the states of residence and injury occurrence are both participating NVDRS states, the state of injury occurrence is responsible for collecting the information. By making this differentiation of responsibility, duplicate reporting is avoided.

† The name of the Institute of Medicine was changed to the National Academy of Medicine, effective July 1, 2015.

§ To be included in NVDRS, deaths of undetermined intent must have some evidence of the possibility that the intent was purposeful, including use of a weapon or other evidence that force was used to inflict the injury. Most commonly, the coroner or medical examiner is unsure whether the death was a suicide or unintentional.

¶ California began collecting data in 2005 but ended data collection in 2009. In 2019, 30 California counties (Amador, Butte, Colusa, Fresno, Glenn, Humboldt, Imperial, Kern, Kings, Lassen, Lake, Los Angeles, Marin, Modoc, Mono, Orange, Placer, Sacramento, San Benito, San Francisco, San Mateo, Santa Cruz, Shasta, Siskiyou, Solano, Sonoma, Tehama, Trinity, Ventura, and Yolo) contributed data to NVDRS.

** Drug trade is defined as the buying, selling, or passing of drugs from one person to another in exchange for goods or money.
